# Ameliorative effect of *Berberidis radix* polysaccharide selenium nanoparticles against carbon tetrachloride induced oxidative stress and inflammation

**DOI:** 10.3389/fphar.2022.1058480

**Published:** 2022-11-09

**Authors:** Fei Gao, Huimin Liu, Hao Han, Xin Wang, Lihua Qu, Congmin Liu, Xuemei Tian, Ranran Hou

**Affiliations:** ^1^ College of Chemistry and Pharmaceutical Sciences, Qingdao Agricultural University, Qingdao, China; ^2^ Agricultural Bio-Pharmaceutical Laboratory, Qingdao Agricultural University, Qingdao, China; ^3^ College of Veterinary Medicine, Qingdao Agricultural University, Qingdao, China; ^4^ Shandong Provincial Key Laboratory of Applied Mycology, Qingdao Agricultural University, Qingdao, China

**Keywords:** *Berberidis radix* polysaccharide, selenium nanoparticles, oxidative stress, hepatoprotection, anti-inflammation

## Abstract

*Berberidis radix* polysaccharide (BRP) extracted as capping agents was applied to prepare BRP-selenium nanoparticles (BRP-SeNPs) in the redox reaction system of sodium selenite and ascorbic acid. The stability and characterization of BRP-SeNPs were investigated by physical analysis method. The results revealed that BRP were tightly wrapped on the surface of SeNPs by forming C-O⋯Se bonds or hydrogen bonding interaction (O-H⋯Se). BRP-SeNPs presented irregular, fragmented and smooth surface morphology and polycrystalline nanoring structure, and its particle size was 89.4 nm in the optimal preparation condition. The pharmacologic functions of BRP-SeNPs were explored *in vitro* and *in vivo*. The results showed that BRP-SeNPs could heighten the cell viabilities and the enzyme activity of GSH-Px and decrease the content of MDA on H_2_O_2_-induced AML-12 cells injury model. *In vivo* tests, the results displayed that BRP-SeNPs could increase the body weight of mice, promote the enzyme activity like SOD and GSH-Px, decrease the liver organ index and the hepatic function index such as ALT, AST, CYP2E1, reduce the content of MDA, and relieve the proinflammation factors of NO, IL-1β and TNF-α in CCl_4_-induced mice injury model. Liver tissue histopathological studies corroborated the improvement of BRP-SeNPs on liver of CCl_4_-induced mice. The results of Western blot showed that BRP-SeNPs could attenuate oxidant stress by the Nrf2/Keap1/MKP1/JNK pathways, and downregulate the proinflammatory factors by TLR4/MAPK pathway. These findings suggested that BRP-SeNPs possess the hepatoprotection and have the potential to be a green liver-protecting and auxiliary liver inflammation drugs.

## 1 Introduction

Liver, the major metabolic and excretory organ in mammals, plays an important role in maintaining homeostasis (Li et al., 2015). Lots of factors such as drugs, viruses, environmental toxicants, alcohol, or metabolic diseases could lead to liver injury (Kanhar and Sahoo, 2019). Carbon tetrachloride (CCl_4_), a representative hepatotoxin, is commonly served as inducement of liver injury model which is used to evaluate the therapeutic potential of drug for liver injury (Song et al., 2021). Numerous studies have shown that the hepatotoxicity of CCl_4_ is the result of oxidative stress and meanwhile it can lead to hepatitis (Che et al., 2019). In liver tissue, CCl_4_ can be catalyzed by cytochrome P450 to form CCl_3_, OOCCl_3_ and reactive oxygen species (ROS) (Liu et al., 2018a). The long-term accumulation of ROS in the body can also lead to various serious chronic liver diseases (Xu et al., 2017). Subsequently, inflammatory cells in liver tissue would be activated by a large number of free radicals, causing liver cell damage and inflammatory response (Faure-Dupuy et al., 2019; Wang W. et al., 2019). At present, the drugs used to treat the liver impairment diseases usually have many side effects and limited curative effect (Wang et al., 2021). It is urgent to find new products to prevent chemical-induced liver oxidative stress damage and relieve liver inflammation.

Selenium is an indispensable micronutrient in living organisms and plays an important role in the prevention of cancer (Sun et al., 2016), diabetes (Liu et al., 2018b), and immune modulation capabilities (Bagheri-Josheghani and Bakhshi, 2022). At same time, selenium is involved in the antioxidant defense systems of the liver and plays an important role in protecting against oxidative stress. Many studies demonstrated that Se supplementation can increase the level of enzymes such as GPx, prevent the accumulation of free radical species, and reduce the cellular damage (Qin et al., 2015). However, the narrow margin between the effective and toxic doses limited the application of this substance. It has been reported that orange-red and zero-valence selenium nanoparticles (SeNPs) have better bioavailability and chemical stability, and are less toxic than other forms of inorganic selenium (Wadhwani et al., 2016). Nevertheless, poor water solubility and the ability to easily transform into a grey analogue, that is, thermodynamically stable but biologically inert, makes Se^0^ difficult to be used in food and medicine fields. Therefore, a kind of material as stabilizer or capping agent is needed for nano-selenium composites. Polysaccharides are rich in hydrophilic groups (such as hydroxyl groups) and can be used as stabilizers to synthesize selenium nanoparticles and improve their stability. Meanwhile, polysaccharides have received extensive attention due to their diverse biological activities and low side-effects (Cheng et al., 2018). Therefore, polysaccharide was selected to synthesize nano-selenium complex, hoping to exert better biological activity and synergistic effect (Huang S. et al., 2020).

*Berberidis radix*, a commonly used Chinese herbal medicine, is the root or stem bark the Berberidaceae, including *Berberis soulieana* Schneid., *Berberis wilsonae* Hemsl., and *Berberis poiretii* Schneid. Or *Berberis vernae* Schneid. And others in the same genus. In traditional Chinese medicine, it is defined bitter in taste and attributive to liver, stomach, and large intestine. It has the function of clearing away heat and draining dampness, and dissipating blood stasis. It is mainly used to treat dysentery, jaundice, pharyngalgia, conjunctival congestion and traumatic injury. *Berberidis radix* polysaccharide (BRP) as the main functional component of *Berberidis radix* may have the similar effect and abundant branch structure and hydroxyl groups, which can be used as stabilizers for the preparation of SeNPs.

In this experiment, we obtained a new polysaccharide from *Berberidis radix*, greenly synthesized and characterizes BRP-SeNPs, and then test the antioxidant activities and anti-inflammation activities *in vitro* and *in vivo*. Therefore, this study aimed to explore whether the *Berberidis radix* polysaccharide-selenium nanoparticles (BRP-SeNPs) have the effect of relieving liver injury and anti-inflammatory activities and its action mechanism, and finally provide a theoretical basis for the research and development of new green liver-protecting and anti-inflammatory drugs.

## 2 Materials and methods

### 2.1 Materials and chemicals

The herbal of *Berberidis radix* was the product from Tibet (China). Sodium selenite (Na_2_SeO_3_), ascorbic acid, hydrogen peroxide (H_2_O_2_) and carbon tetrachloride (CCl_4_) were obtained from Aladdin Industrial Corporation (Shanghai, China). DMEM, FBS, PBS, and Trypsin were purchased from Beyotime Biotechnology Co., Ltd. (Shanghai, China). The ELISA assay kits of AST, ALT, SOD, GSH-Px, MDA, CYP2E1, NO, TNF-α and IL-1β were purchased from Jiangsu Meimian Industrial Co., Ltd. (Yancheng, China). Antibodies of mouse TLR4, p-JNK, p-ERK, p-p38, p-ASK, p-MKK4, Keap1, Nrf2 were purchased from Cell Signaling Technology (Beverly, MA, United States). Antibodies of mouse β-actin and secondary antibody (HRP labeled goat anti-mouse IgG antibody) were purchased from Zhongshan Golden Bridge Biotechnology Co., Ltd. (Beijing, China). In addition, all other chemicals and solvents were analytical grade chemicals and sourced from Sinopharm Chemical Reagent Co., Ltd. (Shanghai, China).

### 2.2 Preparation and characterization of BRP and BRP-SeNPs

#### 2.2.1 Preparation of BRP

50 g *Berberidis radix* power was immersed in 100 ml of 95% petroleum ether solution and refluxed at 60°C for 3 h. The filtered and dried residue was extracted three times with distilled water (1:30 g/ml) at 100°C for 2 h and the filtrates were combined. After that, the filtrate was further condensed to 200 ml with a rotary evaporator at 55°C and then added to four times volume of ethyl alcohol. After standing for 12 h at 4°C, polysaccharides of *Berberidis radix* was precipitated and deproteinated with Sevag reagent (V_N-butyl alcohol_:V_Trichloromethane_ = 1:4). The resulting solution was freeze-dried to obtain the *Berberidis radix* polysaccharide (BRP).

#### 2.2.2 Preparation of BRP-SeNPs

According to the previous reports, the BRP-SeNPs were prepared (Zeng et al., 2019). Briefly, 10 mM sodium selenite was added to prepared BRP (2.0 mg/ml) solution and stirred at 25°C for 30 min. Then, 4 times of fresh ascorbic acid (40 mM) was slowly added in and magnetically stirred for 12 h. The resulting product was dialyzed (MWCO: 8000–14000 Da) with distilled water for 72 h and finally lyophilized to obtain BRP-SeNPs.

#### 2.2.3 Characterization of BRP-SeNPs

The color change of the prepared BRP-SeNPs was recorded by a digital camera (Exterior, EOS 1500D, Canon, Japan). Measurement of mean diameter (particle size), dispersibility index (PDI) and Zeta potential of nanoparticles was used dynamic light scattering (DLS, ZS90, Malvern, United Kingdom). The Fourier transform infrared spectroscopy was obtained by FT-IR spectrometer (FI-IR, NicoletiS10, Thermo Fisher, United States). The UV-Vis spectra were scanned from 200 to 800 nm using UV-Vis spectrophotometer (UV, V-3900, HITACHI, Japan). The XPS spectra of BRP and BRP-SeNPs were analyzed by X-ray electron spectrometer (XPS, AXIS SUPRA+, Shimadzu, Japan). The morphology of BRP and BRP-SeNPs was observed by scanning electron microscopy (SEM, Quanta60, FEI, United States) and transmission electron microscopy (TEM, Talos F200S, FEI, United States). The composition of the samples was analyzed using STEM-HAADF detector and EDX equipped with a transmission electron microscope (HRTEM, Talos F200S, FEI, United States).

### 2.3 Antioxidant activity of BRP-SeNPs *in vitro*

#### 2.3.1 Cell culture

The normal mouse hepatocytes AML-12 cell line (CRL-2254) used herein was purchased from the ATCC. The cells were cultured in DMEM/F12 containing 10% FBS, 100 μg/ml streptomycin and 100 U/mL penicillin in cell incubator (5% CO_2_, 37°C). 100 μL of AML-12 cell suspension (2 × 10^5^ cells/mL) was inoculated in a 96-well plate for 12 h.

#### 2.3.2 Establishment of cells injury model induced by H_2_O_2_

AML-12 cells were treated with different concentrations of H_2_O_2_ (0, 25, 50, 100, 150, 200, 250 μM) for 4 h to induce oxidative injure. After that, 10 μL of CCK-8 was added to each well, and the cells were continued to incubate for 2 h at 37°C. The absorbance value of each well was determined at 450 nm by microplate reader (OD, MD/Spectra Max M2e, Molecular Devices, United States).

#### 2.3.3 Cytotoxicity and protective assays of BRP-SeNPs

AML-12 cells were treated with different concentrations of BRP-SeNPs (0, 50, 100, 200, 300, 400, 500 μg/ml) for 24 h. The cytotoxicity was detected by CCK-8 assays.

In the protection assay, AML-12 cells were cultured with different concentrations of BRP-SeNPs (100, 200, 400 μg/ml) for 24 h and then treated with 100 μM H_2_O_2_ for 4 h. The protective function of BRP-SeNPs was determined by CCK-8 assays.

#### 2.3.4 Changes of intracellular GSH-Px and MDA content

According to the previous procedure, AML-12 cells were cultured in 6-well plates, and then treated with or without BRP-SeNPs and H_2_O_2_. The cells were collected and lysed, and centrifuged to obtain the supernatant. The enzyme activity of GSH-Px and content of MDA was determined according to the manufacturer’s instructions.

### 2.4 Animal treatment and dosage regimen

SPF Kunming mice (female, 20 ± 2 g) were purchased from Jinan Pengyue Laboratory Animal Breeding Co., Ltd. (Shandong, China). Animals were reared under the conditions of standard 12-h light, ambient temperature of 25 ± 2°C, and relative humidity of 60 ± 5%. After 3 days of acclimatization, all animals were used for experiments. All experimental protocols strictly followed the Qingdao Agricultural University guidelines for the care and use of laboratory animals.

25 mice were randomly divided into 5 groups (*n* = 5). Blank control group (BC), model group (MG), positive control group (PC), high dose groups (H) and low dose groups (L). The mice in the H, L groups were intragastric administration with BRP-SeNPs (200, 50 mg/kgbw) and PC group with Silymarin (200 mg/kgbw) for 21 days. The mice in the BC and MG groups were given the same volume of distilled water at the same time. All mice, except BC group, were intraperitoneally injected with 0.2% CCl_4_ (V_Carbon tetrachloride_/V_Olive oil_) at a dose of 0.1 ml/10 gbw after the last intragastric administration, and the BC group mice were intraperitoneally injected with the same volume of olive oil. After a 12 h fast, all mice were sacrificed under anesthesia. Blood samples were collected from the orbits, and serum was harvested by centrifugation (3000 rpm, 10 min, 4°C). The liver was washed with 0.9% normal saline and used for biochemical index determination, histopathology analysis and Western blot analysis.

### 2.5 Biochemical index determination

Biochemical analysis of serum was performed, and serum ALT and AST were detected according to the manufacturer’s instructions.

The liver samples with saline solution were homogenized at a ratio of 1:9 (g/ml). The supernatant was obtained by centrifugation at 8000 rpm for 10 min. Liver oxidation index was determined using SOD, GSH-Px, MDA and CYP2E1 Elisa kits, and inflammation index was tested using NO, TNF-α, IL-1β Elisa kits. All Elisa assays were performed according to the kit’s instructions.

### 2.6 Histopathology analysis

A part of fresh liver was cut and fixed in 4% paraformaldehyde solution and stored for 48 h. After dehydration and transparency, the tissues were embedded in wax block, cut into 5 μm slices and stained with H&E assay.

### 2.7 Western blot analysis

Liver sample was added into 1 mM PMSF (Phenylmethylsulfonyl fluoride) (10 mg: 100 uL). The tissue homogenate was centrifugated (12,000 g, 4°C) for 5 min to obtained the supernatant. The concentration of protein in the supernatant was detected by BCA kit, and then denaturized with the Western-IP loading buffer at 100°C for 5 min to finally get protein sample.

Denatured proteins were separated on 10% sodium lauryl sulfate-polyacrylamide gel electrophoresis (SDS-PAGE) and moved to the membranes of polyvinylidene fluoride (PVDF) (Millipore, United States). After that, the PVDF was blocked with 5% BSA-TBST (Tris Buffered Saline Tween) solution (v/v) for 1 h and then incubated with the corresponding antibodies at 4°C overnight. The membranes were washed with TBST. After washing with TBST, the membranes were incubated with an HRP-IgG antibody at room temperature for 4 h. Then, the membranes were washed three times with TBST. Finally, images were got using a chemiluminescence imaging system and analyzed by ImageJ software.

### 2.8 Statistical analysis

All data were repeated three times and data analysis was performed using SPSS 23.0 analysis software. Data were expressed as Mean ± SD, analyzed by one-way analysis of variance (ANOVA) and Duncan’s multiple range test.

## 3 Results and discussion

### 3.1 Preparation of BRP-SeNPs

**FIGURE 1:**
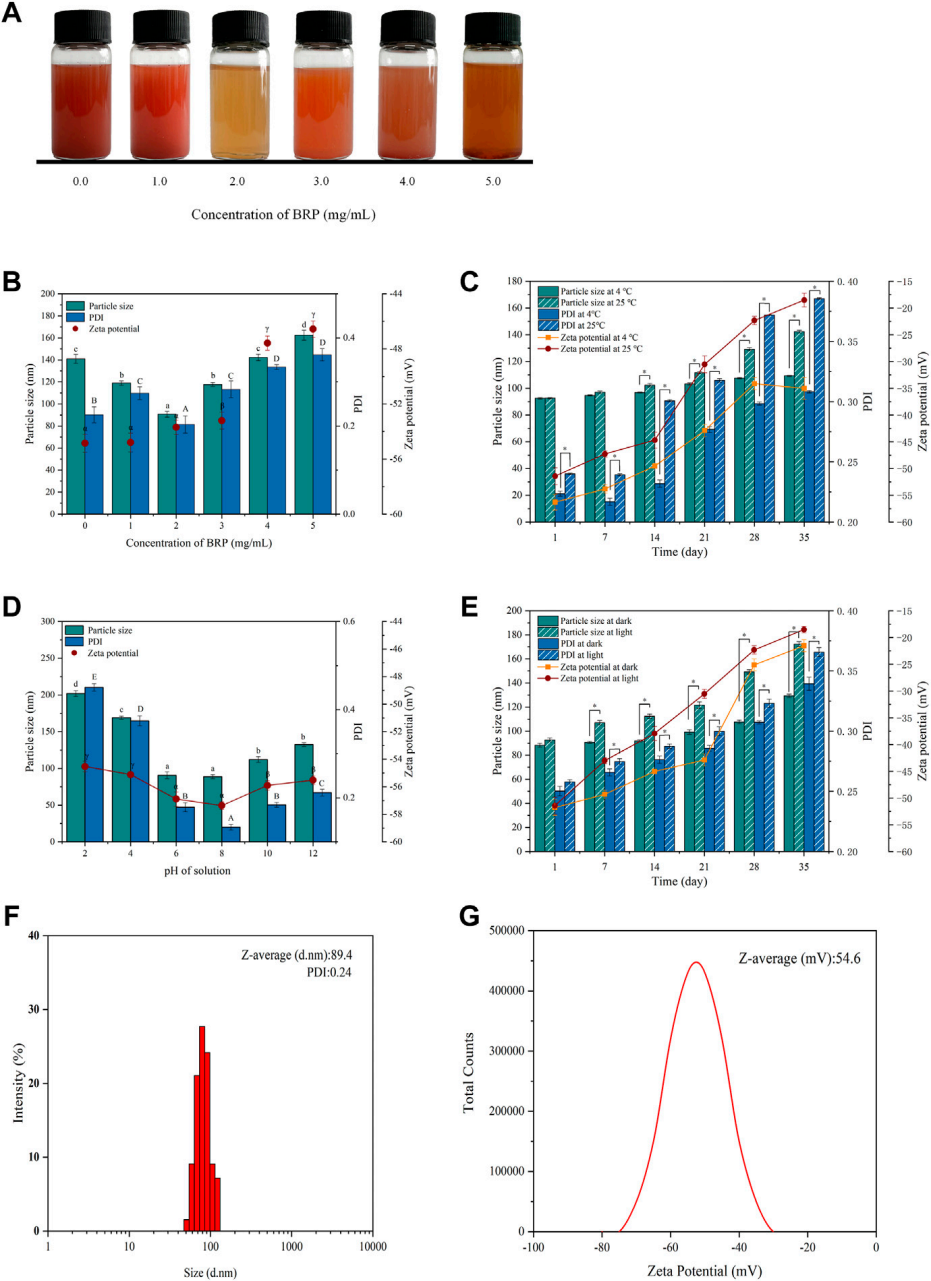
**(A)** Selenium nanoparticles synthesized with different concentrations BRP. **(B)** The influence of BRP concentration on the particle size, PDI and zeta potential of SeNPs. The influence of **(C)** temperature, **(D)** pH and **(E)** light conditions on the stability of SeNPs during 35 days of storage. **(F)** The particle size, PDI and **(G)** Zeta potential of BRP-SeNPs. The different letters indicate significant differences (*p* < 0.05). Comparisons between experimental groups were conducted by using one-way ANOVA, *p < 0.05.

As shown in Figure 1A, Na_2_SeO_3_ was added to the BRP solution and mixed well. The reduction reaction of the precursor SeO_3_^2-^ to selenium (Se) atoms was triggered when ascorbic acid was added, resulting in the formation of SeNPs stabilized by BRP macromolecules. The synthesis process of BRP-SeNPs could be clearly detected by monitoring the dark red color-light yellow or reddish brown. When the polysaccharide concentration was 0 mg/ml to 1.0 mg/ml, the solution was dark red and turbid, and a lot of precipitates were formed. However, when the polysaccharide concentration reached 2.0 mg/ml, the clear and bright orange-red solution was obtained. And then as the polysaccharide concentration increased, the color of the solution gradually brightly deepened.

### 3.2 Stability of BRP-SeNPs

The stability and the size of BRP-SeNPs are always important to affect their biological activity. Therefore, BRP-SeNPs need to be experimentally determined to screen the optimal synthesis conditions. As shown in Figure 1B, the concentration of BRP affected the particle size, PDI and Zeta potential of BRP-SeNPs. When the BRP concentration was 2.0 mg/ml, the particle size (89.4 nm) and PDI (0.24) of synthesized BRP-SeNPs were significant smaller than other groups. The results demonstrated that BRP-SeNPs had comparatively centralized distribution of the particles diameter when BRP used at 2.0 mg/ml. Comparing to the concentration of BRP in 4.0–5.0 mg/ml, the zeta potential of BRP-SeNPs prepared with BRP in 0–3.0 mg/ml were significant higher. The results meant that BRP-SeNPs possessed high Zeta potential had low aggregation and high stability. However, some studies reported that with the increasing of concentration of polysaccharide, the particle size of synthetic polysaccharide-SeNPs would get smaller (Chen et al., 2022). We speculated that the strong molecular interaction between polysaccharides was likely to lead to the weakened binding ability of BRP to SeNPs in our research. The results in Figure 1B demonstrated that the particle size and stability of SeNPs can be successfully regulated by adjusting the concentration of BRP. On this basis, the storage time was extended to 35 days to study the potential effects of temperature, pH, and light on the stability of BRP-SeNPs.

As shown in Figure 1C, when the solution was stored at 25°C, the particle size of BRP-SeNPs increased significantly from 92.6 to 138.4 nm in 35 days, while there was only varied 16.5 nm (92.6–109.1 nm) at 4°C during the same time. The changes of PDI and Zeta potential of BRP-SeNPs were dramatically increased after 14 days whether it was stored at 4°C or 25°C. Comparing with the BRP-SeNPs stored at 25°C, the PDI and Zeta potential of BRP-SeNPs stored at 4°C were significant lower. Those results indicated that the BRP-SeNPs stored at 25°C had poor stability. The reason may be that the surface charge of spherical SeNPs is weakened by the high temperature, which reduces the stability and makes the SeNPs nanoparticles turbid and unstable (Song et al., 2020).

As shown in Figure 1D, pH also had a significant effect on particle size and PDI. It was found that the particle size of BRP-SeNPs increased significantly and was unstable under acidic conditions. The reason might be that BRP is protonated under strongly acidic conditions, which weakens the electrostatic interaction between BRP and SeNPs, resulting in the aggregation of SeNPs. In contrast, under weakly alkaline conditions, the particles of the BRP-SeNPs solution have less aggregation and better stability.

In addition, as shown in Figure 1E, the effects of light conditions (500 ± 40 Lx) on the physicochemical stability of BRP-SeNPs were also determined. The results of particle size and PDI slightly increased when the storage time was extended to 35 days under dark conditions. In contrast, under illumination, the particle size of BRP-SeNPs significantly increased. This phenomenon may be due to the photosensitivity of the O-H which likely conjugated to the SeNPs, thereby affecting the stability of the solution (Gao et al., 2020). In order to obtain BRP-SeNPs with smaller size and better stability, the optimal synthesis and storage conditions were as follows. The reaction system contained 10 mM, 2.0 mg/ml BRP, and 40 mM ascorbic acid. The solution system could store in 4°C without light. Under these conditions, the particle size of BRP-SeNPs was 89.4 nm, the PDI was 0.24, and the Zeta potential was −54.6 mV, as shown in Figures 1F,G.

### 3.3 Characterization of BRP-SeNPs

**FIGURE 2:**
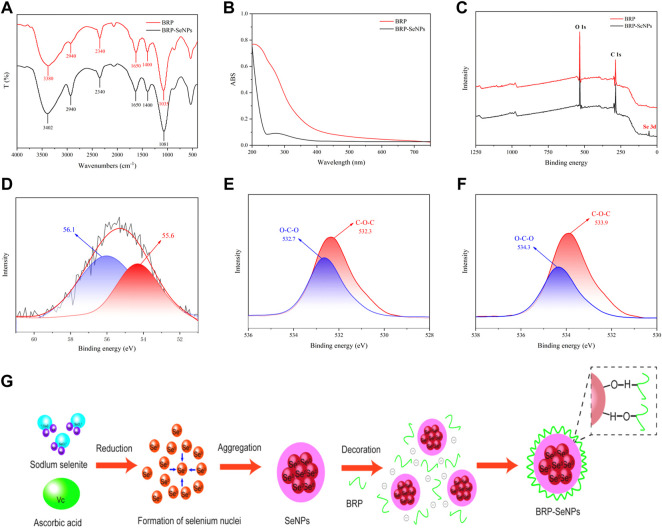
Characterization of BRP-SeNPs. **(A)** FT-IR spectrum. **(B)** UV-vis absorption spectrum. **(C)** XPS spectrum of BRP and BRP-SeNPs. **(D)** High-resolution Se 3 days spectrum of BRP-SeNPs. O1s spectrum of **(E)** BRP and **(F)** BRP-SeNPs. **(G)** Schematic diagrams of formation mechanism of BRP-SeNP.

The BRP-SeNPs were further characterized by FT-IR spectroscopy. As shown in Figure 2A, the peak of BRP at 3380 cm^−1^ represented the stretching vibration of the hydroxy group, which slightly shifted to 3402 cm^−1^ in the IR spectrum of BRP-SeNPs (Wang L. et al., 2019). The sharp band at 2940 and 2340 cm^−1^ were the C-H stretching vibration in methylene group and carbon dioxide peak in the testing environment (Shi et al., 2021). The absorption band at 1650 cm^−1^ could be assigned to the absorption of water, and the absorption peaks at 1400 cm^−1^ represented the bending vibration of -OH (Zhang et al., 2021). Meanwhile, the peak of BRP at 1035 cm^−1^ was caused by the stretching vibration of C-O-C and C-O-H, which slightly shifted to 1081 cm^−1^ in the IR spectrum of BRP-SeNPs (Xiao et al., 2017). Moreover, the characteristic band of BRP-SeNPs at 3402 cm^−1^ was relatively lower than that of BRP (3380 cm^−1^), with an obvious blue shift (Liu et al., 2012), indicating that the hydroxyl groups of BRP were bound by hydrogen bonding (O-H⋯Se) between the surface atoms of SeNPs (Zhang et al., 2015). In addition, the absorption band of C-O-H of BRP-SeNP appeared at higher wavelength at 1081 cm^−1^ (Liu et al., 2012) compared with the BRP, indicating that some hydroxy groups of BRP were conjugated with SeNPs to disrupt hydrogen bond in native BRP and formed new C-O⋯Se bonds (Liu et al., 2017; Hui et al., 2019). FT-IR spectroscopy showed the interaction mechanism between BRP and SeNPs. As shown in Figure 2B, UV-Vis absorption spectrum of BRP and BRP-SeNPs was detected in the range of 200–800 nm. The UV-Vis spectrum of BRP did not exhibit obvious absorption peaks between 200 and 400 nm, and thus illustrated its contained minimal proteins and nucleic acids. However, the UV-Vis spectrum result of BRP-SeNPs showed an obvious absorption peak near 270 nm and that explained the formation of SeNPs and the interaction between BRP and SeNPs which had been werified in previous results (Zhai et al., 2017).

In order to explain the interaction mechanism between BRP and SeNPs, XPS was used to further explore. As shown in Figure 2C, there was a typical Se 3 days peak in the full spectrum of BRP-SeNPs compared with BRP, indicating that the selenium was successfully introduced into BRP-SeNPs. In addition, according to the NIST X-ray photoelectron spectroscopy database (standard reference database 20, version 4.1) and the high-resolution of Se 3 days spectrum of BRP-SeNPs, two dominant peaks at 56.1 eV (Se 3d_3/2_) and 55.6 eV (Se 3d_5/2_) were observed in Figure 2D, indicating the selenium in the BRP-SeNPs was zero valence selenium (Se^0^) (Gao et al., 2020). Besides, the Se 3 days spectrum of BRP-SeNPs had no electron binding energy peak at 59.1 eV, indicating that all Se^4+^ were reduced to Se^0^ in this reaction system (Jiang et al., 2022).

As shown in Figures 2E,F, the O1s spectra of BRP and BRP-SeNPs displayed that the binding energy was increased from 532.7 to 532.3 eV in BRP to 534.3 and 533.9 eV in BRP-SeNP, which might be attributed to the strong interactions and the formation of C–O⋯Se bonds between SeNPs and BRP (Ren et al., 2021). The results mentioned-above indicated that the strong interaction between BRP and SeNPs during the formation of BRP-SeNPs reduced the electron density around the O atom, which in turn weakened the shielding effect on the inner electrons, making the O atom’s binding energy increase and the formation of C-O⋯Se bonds (Cai et al., 2018). This result of the formation of C-O⋯Se bonds in BRP-SeNPs was consistent with that showed in FT-IR analysis. Combined with all results, the formation mechanism of BRP-SeNPs was showed in Figure 2G. In the reaction system, free Se^4+^ (SeO_3_^2-^) was firstly reduced to form Se^0^ by ascorbic acid, then the original Se core was subsequently generated and amount of Se^0^ finally aggregated into SeNPs. Next, BRP wrapped on the surface of SeNPs through the formation of C-O⋯Se bonds or the strong interaction by the hydroxyl group between BRP and SeNPs to produce BRP-SeNPs.

**FIGURE 3:**
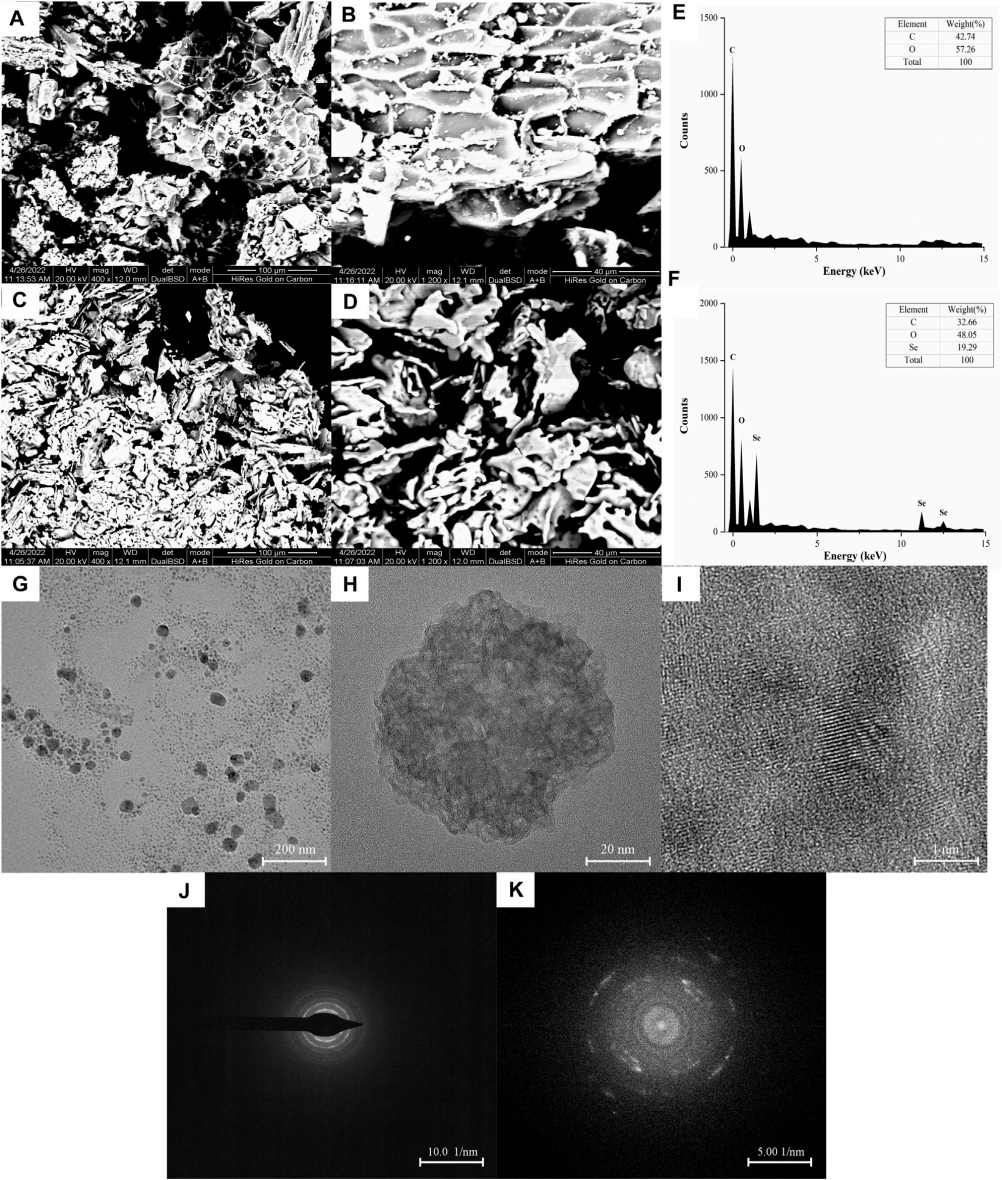
SEM images of **(A,B)** BRP and **(C,D)** BRP-SeNPs, A and C under ×400 magnification, B and D under 1200 × magnification. The distribution of C, O, and Se in **(E)** BRP and **(F)** BRP-SeNP by EDX analysis. Representative **(G)** TEM and **(H,I)** HRTEM images of BRP-SeNPs and its corresponding **(J,K)** SAED pattern.

The surface morphology and elemental composition of BRP-SeNPs were analyzed by SEM and EDX techniques. As shown in Figures 3A–F, BRP presented areatus, foveolate and rough surface morphology. However, BRP-SeNPs revealed irregular, fragmented and smooth surface morphology. Then we determined the three elements of C, O, and Se in BRP and BRP-SeNPs. The results showed that C and O in BRP were 42.74% and 57.26%, and C, O, and Se in BRP-SeNPs were 32.66%, 48.05% and 19.29%, respectively. After that, a small amount of BRP-SeNPs solution was placed on a copper grid and dried, and then observed by transmission electron microscopy (Zhang et al., 2014). As shown in Figure 3G, the BRP-SeNPs presented well-dispersed spherical particles with a diameter of about 60 nm. Interestingly, the size of particle measured by DLS was bigger than that by TEM, which is due to that DLS measured the size of the polysaccharide-conjugated selenium nanoparticles while TEM only observed SeNPs without polysaccharides visualization which supports the preceding research result (Bai et al., 2020). Under the observation of HRTEM, as shown in Figures 3H,I, there were a lot of crystal structures on the spherical surface of BRP-SeNPs. After selected area electron diffraction (SAED) analysis, as shown in Figures 3J,K, bright light bands could be clearly seen, which appeared as circular aperture, indicating that the BRP-SeNPs possessed polycrystalline nanoring structures.

### 3.4 Antioxidant activities of BRP-SeNPs *in vitro*

#### 3.4.1 Effects of H_2_O_2_ and BRP-SeNPs on the viability of AML-12 cells

**FIGURE 4:**
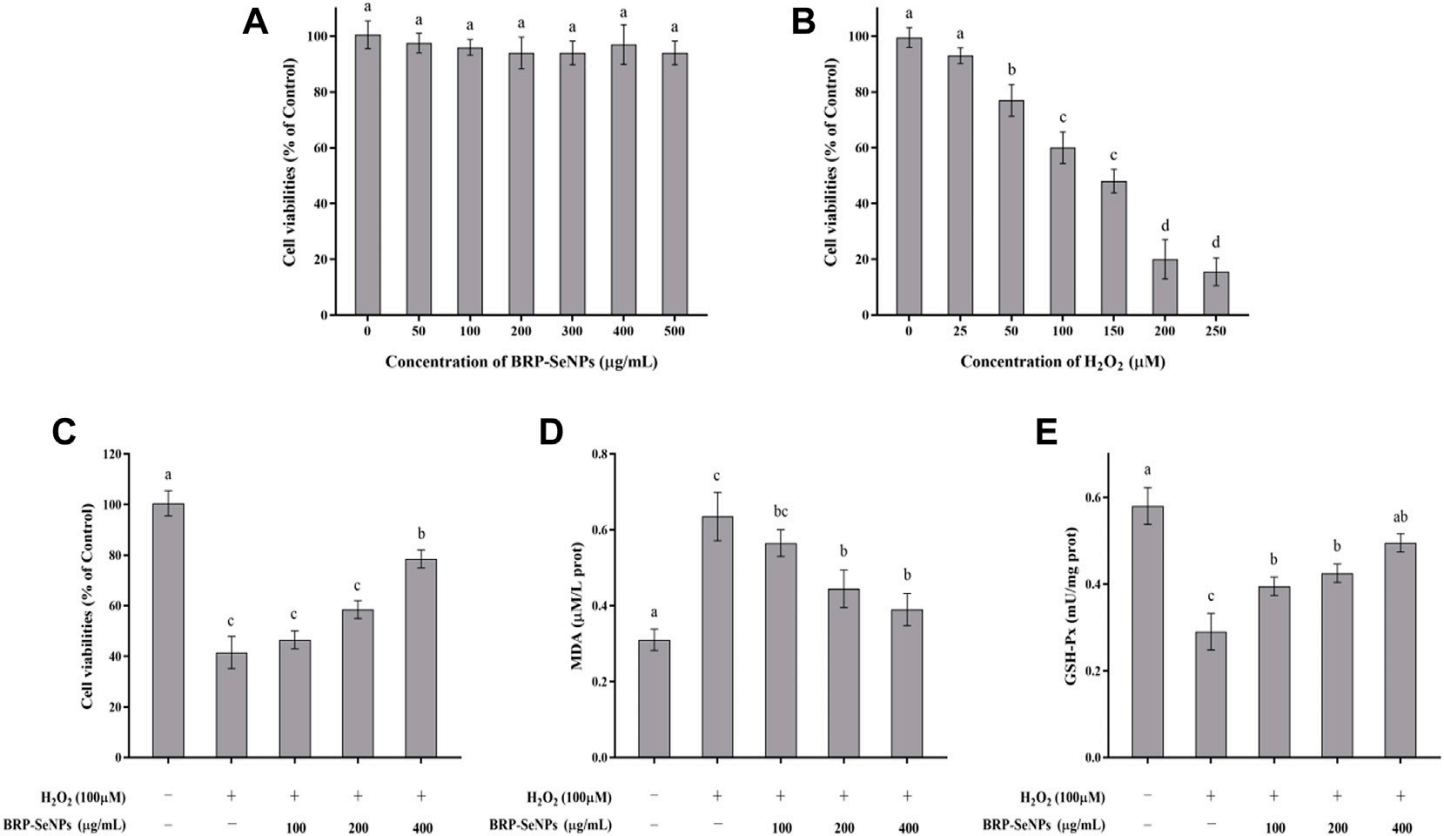
**(A)** Evaluation of toxicity of BRP-SeNPs on AML-12 cells. **(B)** Test of H_2_O_2_ concentration on AML-12 cells. **(C)** Protective effect of BRP-SeNPs on the H_2_O_2_-induced AML-12 cells model. **(D)** Effect of BRP-SeNPs on the level of MDA on H_2_O_2_-induced AML-12 cells model. **(E)** Effect on the activity of GSH-Px on H_2_O_2_-induced AML-12 cells model. The different letters indicate significant differences (*p* < 0.05).

The effect of BRP-SeNPs on the viability of AML-12 cells was detected by CCK-8 method. As shown in Figure 4A, when AML-12 cells were stimulated with different concentrations of BRP-SeNPs, the cell viabilities of BRP-SeNPs groups had no significant difference compared with the BC group. It indicated that BRP-SeNPs had no toxic effect on AML-12 cells in the concentration range of 0–500 μg/ml. Therefore, BRP-SeNPs at concentrations of 100, 200, and 400 μg/ml were used in subsequent experiments. After that, AML-12 cells were stimulated with 0–250 μM H_2_O_2_ for 4 h, as shown in Figure 4B, the cell viability decreased as with the increase of H_2_O_2_ concentration. When the H_2_O_2_ concentration was 25 μM, there was no significant difference on cell viability. When the H_2_O_2_ concentration reached 100 μM, the cell viability was 60.30 ± 2.39% of BC group, and the cell was in a reversible state. Therefore, we selected 100 μM of H_2_O_2_ to stimulate AML-12 cells for 4 h as cellular oxidative injury model.

#### 3.4.2 Effects of BRP-SeNPs on H_2_O_2_-induced oxidative injury model

AML-12 cells were pretreated with BRP-SeNPs at 0, 100, 200, 400 μg/ml for 24 h, and then stimulated with H_2_O_2_ for 4 h. As shown in Figure 4C, after treatment with 100, 200, and 400 μg/ml of BRP-SeNPs, the cell viability was significantly higher than that in the MG group in a dose-dependent manner. This indicated that BRP-SeNPs could alleviate the decrease in cell viability induced by H_2_O_2_.

The effect of BRP-SeNPs on MDA production was shown in Figure 4D. Compared with the BC group, the MDA content of the cells stimulated by H_2_O_2_ was significantly increased. However, BRP-SeNPs pretreatment significantly decreased the MDA content of H_2_O_2_-induced AML-12 cells. The results showed that BRP-SeNPs could markedly reduce intracellular MDA production.

The changes of intracellular GSH-Px activity during H_2_O_2_-induced cell injury were shown in Figure 4E. GSH-Px is an antioxidant enzyme that catalyzes the decomposition of hydrogen peroxide. Compared with the BC group, the GSH-Px activity of H_2_O_2_-induced AML-12 cells was significantly decreased, however, BRP-SeNPs restored the GSH-Px activity and even showed no significant difference at 400 μg/ml compared with BC group. This suggested that BRP-SeNPs may recover AML-12 cells by protecting against H_2_O_2_-induced reduction in antioxidant enzyme activity.

### 3.5 Effect of BRP-SeNPs on body weight and relative organ index *in vivo*

**FIGURE 5:**
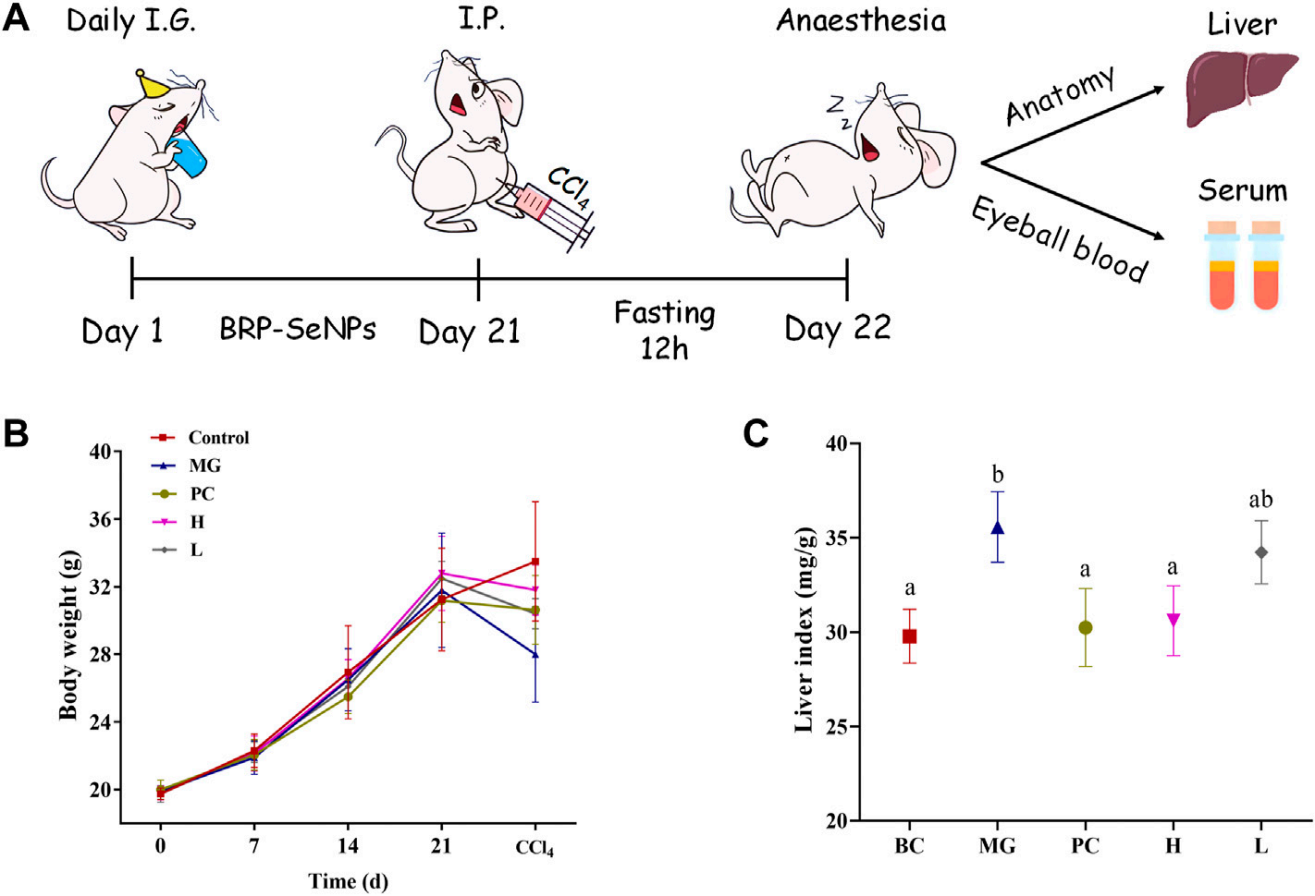
**(A)** Animal experiment process in mice. **(B)** Effect of BRP-SeNPs on body weight of mice. **(C)** Effect of BRP-SeNPs on organ index in CCl_4_-induced mice. The different letters indicate significant differences (*p* < 0.05).

Animal experiment process in mice as shown in Figure 5A. The effect of BRP-SeNPs on the body weight of mice was shown in Figure 5B. During the first 3 weeks, the average body weight of each group in the natural growth state gradually increased. Compared with the BC group, the MG, PC, and BRP-SeNPs groups had no significant effect on the body weight of the mice. After the injection with CCl_4_ and fasting treatment, the body weight of mouse was obviously decreased. The weight loss rate of H group and L group was lower than that of MG group. It revealed that the mice pretreated with BRP-SeNPs possessed stronger protective functions against CCl_4_-induce damage. The liver index of the MG group was significantly higher than that of the BC group (Figure 5C), indicating that CCl_4_ could cause changes in the liver, loss its function and even clog in the organ. The liver indexes of mice treated with BRP-SeNPs were lower than that of MG group, and H group was significantly lower than that of MG group. The results suggested that BRP-SeNPs may promote the function of liver and gain the weight body to protect against injury induced by CCl_4_.

### 3.6 Liver histopathological

**FIGURE 6:**
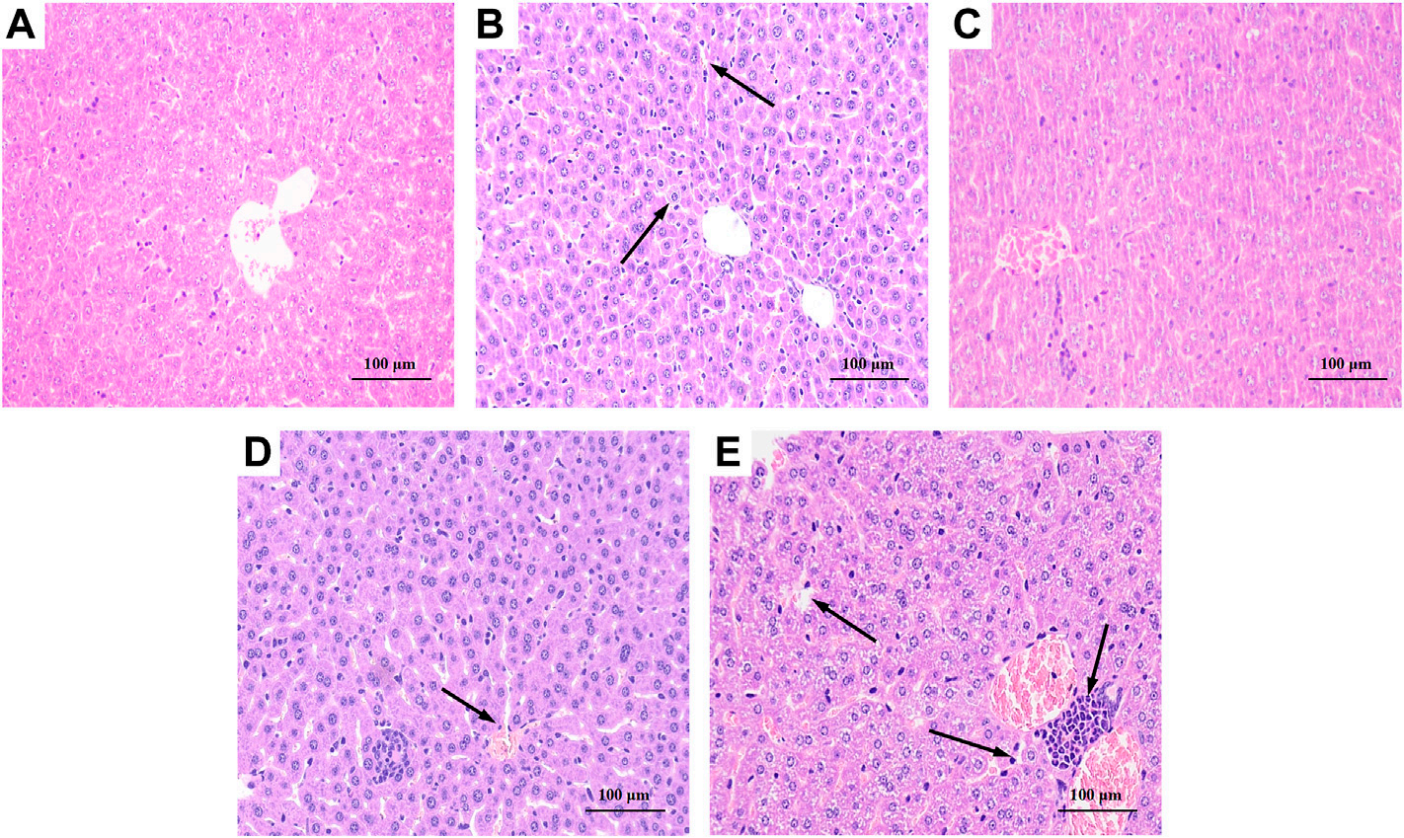
Effect of BRP-SeNPs on liver histopathological changes in CCl_4_-induced mice (×200 Magnification). **(A)** BC, **(B)** MG, **(C)** PC, **(D)** H, and **(E)** L group. Areas of severe histopathological changes were marked by black arrows.

As shown in Figure 6A, the liver tissue of the BC group was observed after staining, and the hepatocytes were arranged regularly and the morphology was normal. The liver tissue of the MG group, as shown in Figure 6B, exhibited hepatocyte degeneration, blurred cell boundaries, inflammatory cell infiltration, plasma vacuolization, hepatocyte necrosis, and all that indicated that intraperitoneal injection of CCl_4_ would cause severe damage to the liver of mice. Figure 6C showed that the liver of PC basically returned to a normal state except for loose arrangement. As shown in Figures 6D,E, BRP-SeNPs pretreatment could significantly resist the invasion and damage of the liver by CCl_4_-induced histopathological changes, especially for the high-dose group.

### 3.7 Biochemical assays detected in blood serum

**FIGURE 7:**
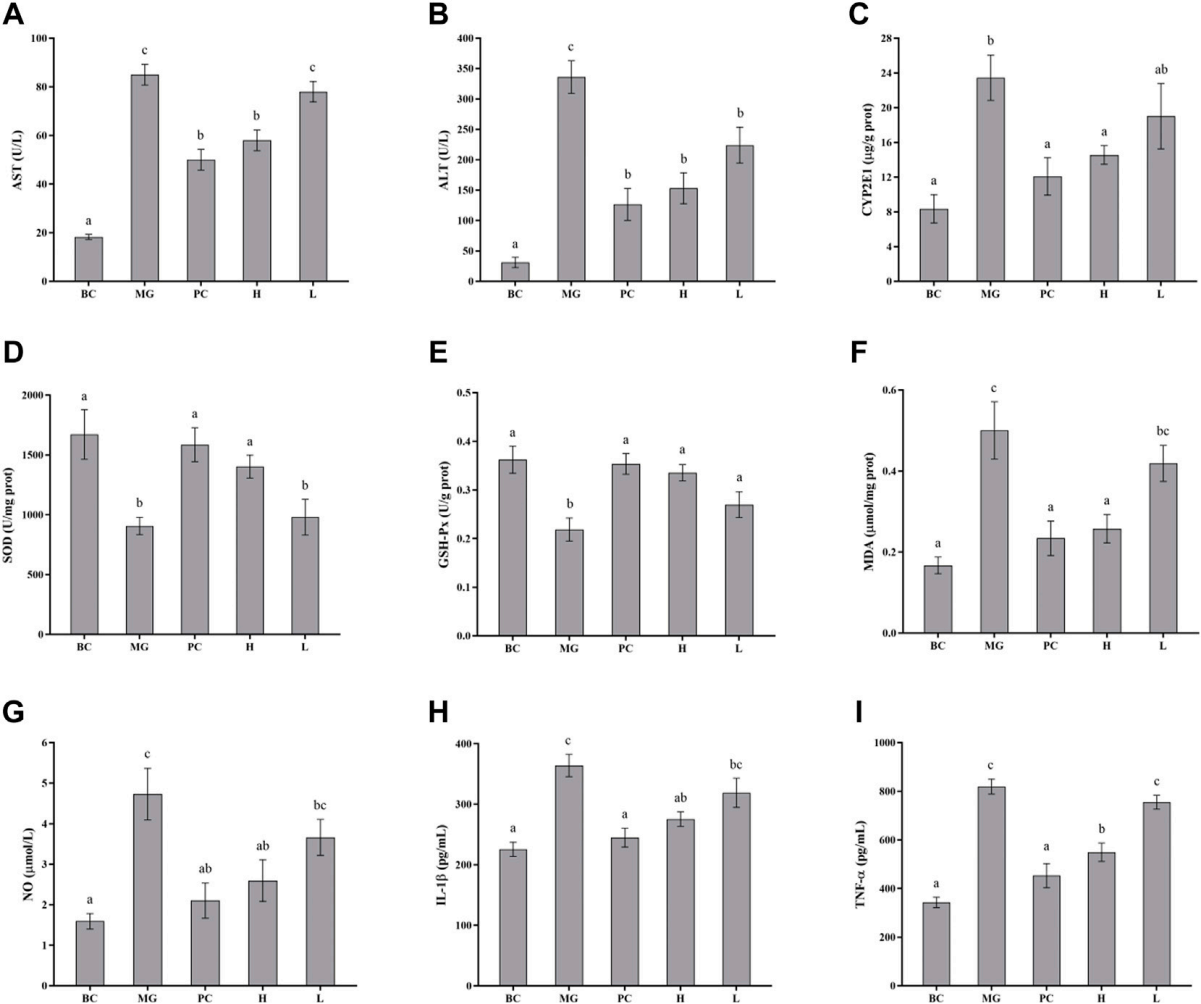
Effect of BRP-SeNPs on the contents of **(A)** AST, and **(B)** ALT in serum of mice induced by CCl_4_. **(C)** Represents the content of CYP2E1 in the liver. **(D–I)** Effect of BRP-SeNPs on indicators of liver oxidative stress and inflammatory mediators induced by CCl_4_. **(D–F)** Represent the content of SOD, GSH-Px and MDA, and **(G–I)** represent the content of NO, IL-1β and TNF-α in the liver, respectively. The different letters indicate significant differences (*p* < 0.05).

The effects of BRP-SeNPs on serum marker levels in mice were demonstrated in Figures 7A,B. The levels of serum ALT and AST in the MG group were significantly higher than that in the BC group. After the mice treated with different concentrations of BRP-SeNPs for 21 days and induced by CCl_4_, the serum AST and ALT levels were decreased to a certain extent. Concretely speaking, the serum AST levels of H groups was significantly lower than MG and the serum ALT levels of L and H groups were significantly reduced compared with MG. The serum parameters indicated that BRP-SeNPs could improve the function and activity of hepatocyte stimulated by exogenous injury-inducing factor to suppress the release of ALT and AST.

### 3.8 CYP2E1 changes in liver

CYP2E1 is a major drug metabolizing enzyme, and its overexpression in the liver can lead to oxidative stress (Song et al., 2011). As shown in Figure 7C, the CYP2E1 enzyme content in the MG group was significantly higher than BC group (*p* < 0.05), and it confirmed that CCl_4_ caused significant increase in the expression of CYP2E1 enzyme in the liver of mice. Silymarin could restore CYP2E1 levels in the mice induced by CCl_4_. BRP-SeNPs could significantly reduce the rise of CYP2E1 expression induced by CCl_4_ (*p* < 0.05). When the mice were given BRP-SeNPs of 200 mg/kgbw, the CYP2E1 level in liver of the mice induced by CCl_4_ was similar as PC group. This indicated that BRP-SeNPs could enhance the function of liver and restrain the expression of hepatic CYP2E1 to relieve oxidative stress.

### 3.9 Biochemical assays detected in liver

Oxidative damage caused by reactive oxygen species (ROS) is an important part of CCl_4_-induced liver damage. When ROS rise in the body, excess free radicals could lead to lipid peroxidation reaction that damages cell membranes and organic damage. At the same time, ROS also reduced the activity of antioxidant enzymes *in vivo*, resulting in a further increase in oxidative damage. When the liver is damaged by external factors, it will cause a series of stress responses. Thus, we determined the antioxidant enzyme activity such as SOD and GSH-Px, and the content of MDA. Besides, in order to investigate whether BRP-SeNPs have therapeutic effect on the inflammation caused by CCl_4_-induced liver injury, detection kits were also used to test the relative pro-inflammatory cytokines (NO, IL-1β, TNF-α) in the liver.

#### 3.9.1 Effects on SOD and GSH-Px activity and MDA levels in liver

SOD and GSH-Px are antioxidant enzymes that could reduce the oxidation caused by ROS, and play an important role in maintaining ROS balance in the body. As shown in Figures 7D–E, CCl_4_ decreased the SOD and GSH-Px activities in MG compared with the BC group, indicating that CCl_4_ may cause damage to the hepatocyte and further inhibit the express of the two antioxidant enzymes. While the mice administrated with BRP-SeNPs could significantly recover the activities of SOD and GSH-Px in a concentration-dependent manner. This indicated that BRP-SeNPs could strengthen the liver function and the expression of antioxidant enzyme to attenuate the injury induced by CCl_4_.

Hyperoxidation of lipids is a hallmark of oxidative stress. As shown in Figure 7F, compared with the MG group, the content of MDA in H and PC group was significantly decreased and had no significant difference with BC. This indicated that BRP-SeNPs could effectively alleviate CCl_4_-induced lipid peroxidation. So far, we could speculate from the results mentioned above that BRP-SeNPs could improve the liver function, heighten the hepatocellular activity and activate the antioxidant system to relieve the liver injury evoked by exogenous pathogenic factors such as CCl_4_.

#### 3.9.2 Effects on NO, IL-1β and TNF-α levels in liver

As shown in Figure 7G, CCl_4_ could significantly increase the NO production compared with normal mice. However, BRP-SeNPs displayed good inhibitory effect on the NO concentration in a dose-dependent manner. As shown in Figures 7H,I, the levels of IL-1β and TNF-α in BC, PC and H group were significantly lower than that in the MG. All the results of proinflammatory cytokines showed that BRP-SeNPs possessed the anti-inflammatory activity by inhibiting the production of NO, L-1β and TNF-α in CCl_4_-induced liver injury model.

### 3.10 Cell signaling pathway

**FIGURE 8:**
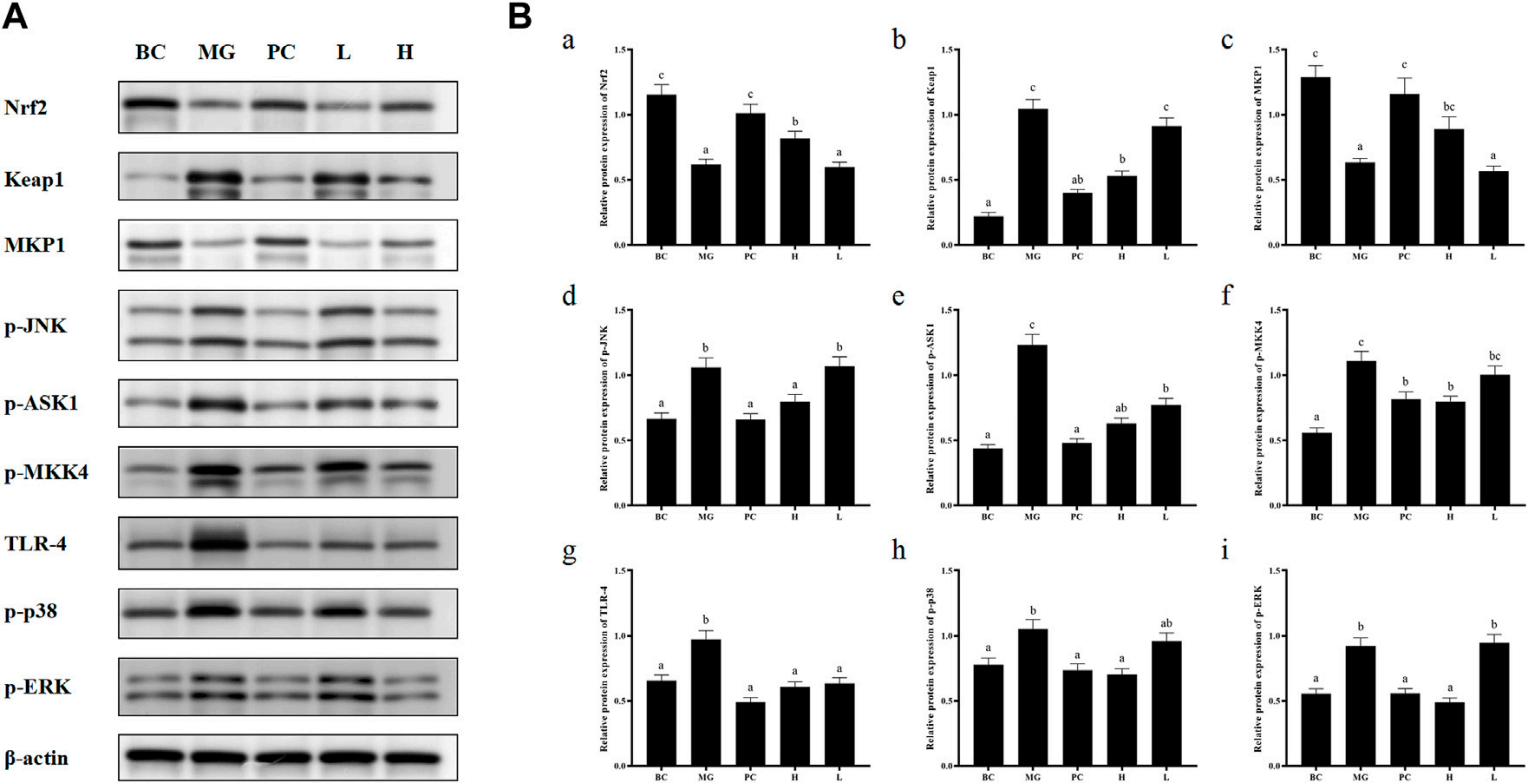
**(A)** BRP-SeNPs protected mice from CCl_4_-induced liver injury *via* Nrf2/Keap1/MKP1/JNK and TLR4/MAPK signaling pathways. **(B)** Quantification of **(A)** Nrf2, **(B)**Keap1, **(C)**MKP1, **(D)** p-JNK, **(E)** p-ASK1, **(F)** p-MKK4, **(G)** TLR4, **(H)** p-p38, **(I)** p-ERK expression in cytoplasm. The different letters indicate significant differences (*p* < 0.05).

Activated Nrf2 transferred into nucleus, interacts with ARE, and induces the expression of downstream targets to regulate oxidative stress (Ning et al., 2018). In general, the liver is a metabolically active organ possessing a wide range of antioxidant systems, and correspondingly, it is likely that oxidative stress plays a key role in promoting the development of acute liver injury. In this experiment, in order to elucidate the molecular mechanism of the protective effect of BRP-SeNPs on the liver and the inhibitory effect on inflammation, we analyzed the expression of Nrf2, Keap1, MKP1, p-JNK, p-ASK1, p-MKK4, TLR4, p-38, p-ERK. Inactive state Nrf2 interacts with actin-binding protein Keap1 in the cytoplasm and is rapidly degraded by the ubiquitin-proteasome pathway. However, when Nrf2 is exposed to oxidative or electrophilic stress, the phosphorylation of Nrf2 leads to the dissociation of the Nrf2-Keap1 complex, and then, stable Nrf2 translocates to the nucleus. In the nucleus, Nrf2 binds to the ARE sequence and promotes the expression of many antioxidant proteins and phase II detoxifying enzymes including GSH-Px and SOD, which are closely related to eliminating excessive free radicals and protecting against oxidative stress. The results showed that BRP-SeNPs attenuated liver injury by upregulating Nrf2 and downregulating Keap1 in the Nrf2/ARE signaling pathway in Figure 8. It was in keeping with the activity changes of SOD and GSH-Px tested in our research and the report (Meng et al., 2021). It was in keeping with the activity changes of SOD and GSH-Px tested in our research. It indicated that BRP-SeNPs could regulate and control Nrf2/ARE pathway, and finally showed hepatoprotective effect on CCl_4_-induced liver injury.

In the anti-inflammatory mechanism, we explored the TLR4/MAPK pathway. CCl_4_ significantly increased the expression of TLR4 protein in mouse liver, and BRP-SeNPs could inhibit CCl_4_-induced up-regulation of TLR4 protein expression in mouse liver. Stimulated TLR4 can activate the MAPK signaling pathway and accordingly lead to the expression of p-p38, p-ERK, and p-JNK protein. Further, they promote the synthesis and release of inflammatory factors. However, it can be seen that BRP-SeNPs can reduce the release of NO, IL-1β and TNF-α by down-regulating the expression of TLR4/MAPK pathway in the liver of mice induced by CCl_4_ in Figure 8.

In the anti-apoptosis mechanism, we determined the ASK1/MKK4/JNK pathway. BRP-SeNPs could inhibit the expression of p-ASK1 and p-MKK4 in the CCl_4_-induced damage. As we all know, JNK plays an important role in the ROS-induced apoptosis, including mitochondrial apoptotic pathway in the cytoplasmic and transcription factor pathway in the nuclear. However, BRP-SeNPs suppressed the expression of p-JNK by ASK1/MKK4 pathway and finally maintained the activity and function of hepatocyte and liver. It was consistent to histopathological changes in our study.

In addition, MKP1 is another important protein in protecting liver injury. It upregulates the expression of Nrf2, interacts directly with Nrf2 and promotes the Nrf2/ARE pathway during hepatoprotection (Wang M. et al., 2019). Meanwhile, MKP1 is an endogenous key hepatoprotective factor, which can upregulate antioxidant pathways and inhibit the activation of JNK during liver injury (Li et al., 2017). High-dose BRP-SeNPs could significantly reduce CCl_4_-induced hepatic JNK-activating protein expression and reverse it to normal levels achieving therapeutic effect. After detecting the expression of MKP1 protein, the results showed that CCl_4_ significantly inhibited the expression of MKP1 in mice liver, and high-dose BRP-SeNPs could increase the expression of MKP1 protein in turn.

**FIGURE 9:**
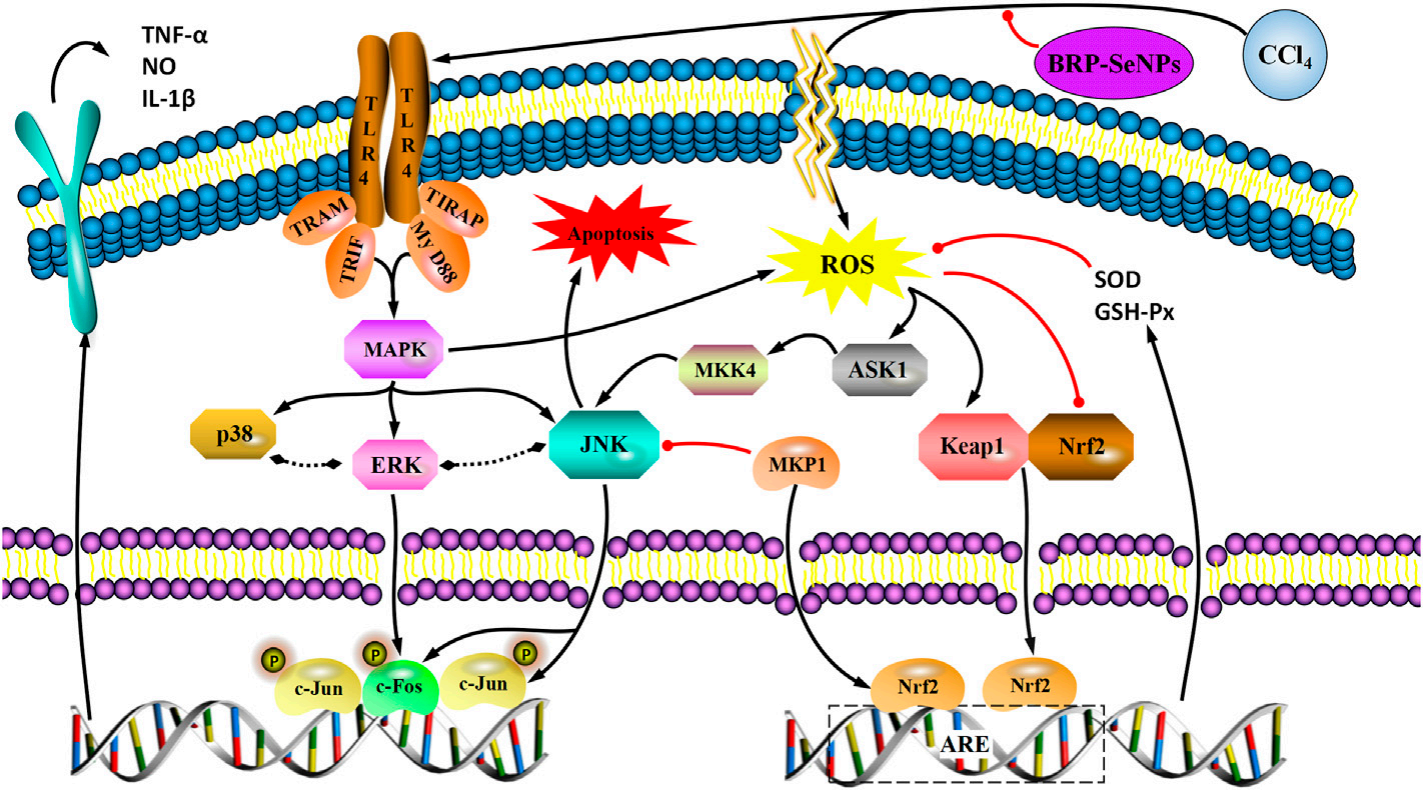
A proposed mechanism for the hepatoprotective effect of BRP-SeNPs on CCl_4_-induced liver injury *in vivo*. Black arrow represents the activation and red line the inhibition.

Taken all the results of mechanism, we found that BRP-SeNPs possessed hepatoprotection by the Nrf2/Keap1/MKP1/JNK pathways, and anti-inflammatory by TLR4/MAPK pathway in Figures 8, 9.

## 4 Conclusion

In summary, *Berberidis radix* polysaccharide (BRP) as a stabilizer obtained from *Berberidis radix* was used to prepare the BRP-selenium nanoparticles (BRP-SeNPs) in the redox reaction system of sodium selenite and ascorbic acid. The stability and characterization of BRP-SeNPs were investigated by DLS, FT-IR, UV-Vis, XPS, SEM, TEM, STEM-HAADF, EDX. The optimal preparation condition was established and the BRP-SeNPs were successfully synthesized. Then, *in vitro* tests, BRP-SeNPs had protective effects on H_2_O_2_-induced AML-12 cells injury model. Subsequently, the preventive effect of BRP-SeNPs on in CCl_4_-induced mice liver injury was explored. BRP-SeNPs could increase the body weight of mice, the activity of SOD and GSH-Px in liver, and meanwhile decrease the liver organ index, ALT and AST in serum, CYP2E1, the content of MDA and the level of NO, IL-1β, and TNF-α in the liver of CCl_4_-induced damage. The histomorphology of liver was restored under the stimulating of BRP-SeNPs. Finally, the hepatoprotective mechanism against CCl_4_-induced liver injury was elucidated. The results indicated that BRP-SeNPs could attenuate oxidant stress by the Nrf2/Keap1/MKP1/JNK pathways, and downregulate the proinflammatory factors by TLR4/MAPK pathway. Hence, BRP-SeNPs possess the hepatoprotection and have the potential to be a green liver-protecting and auxiliary liver inflammation drugs.

## Data Availability

The original contributions presented in the study are included in the article/Supplementary Material, further inquiries can be directed to the corresponding authors.

## Abbreviations

BRP, *Berberidis radix* polysaccharide; BRP-SeNPs, BRP-selenium nanoparticles; GSH-Px, Glutathione peroxidase; MDA, Malondialdehyde; ALT, Alanine aminotransferase; AST, Aspartate aminotransferase; CYP, Cytochrome P450; TLR4, Toll-like receptor 4; p-JNK, Phosphorylation-Jun kinase; p-ERK, Phosphorylation-extracellular regulated protein kinases; p-p38, Phosphorylation-p38; p-ASK1, Phosphorylation-Apoptosis signal-regulating kinase 1; Keap1, Kelch-like ECH-associated protein 1; Nrf2, Nuclear factor erythroid 2-related factor 2; p-MKK4, Phosphorylation-mitogen activated protein kinase 4; ARE, Antioxidant response element; PMSF, Phenylmethylsulfonyl fluoride; SDS-PAGE, Sodium dodecyl sulfate polyacrylamide gel electrophoresis; PVDF, Polyvinylidene fluoride; TBST, Tris Buffered Saline Tween; ROS, Reactive oxygen species; DLS, Dynamic light scattering; FT-IR, Fourier transform infrared spectroscopy; UV-Vis, Ultraviolet-visible spectroscopy; XPS, X-ray photoelectron spectrometer; SEM, Scanning electron microscope; STEM, Scanning transmission electron microscopy; HAADF, High-angle annular dark field; TEM, Transmission electron microscopy; EDX, Energy Dispersive X-Ray Spectroscopy.
