# Supplementary figures and images for: Ameliorative effect of Berberidis radix polysaccharide selenium nanoparticles against carbon tetrachloride induced oxidative stress and inflammation
Source: Front Pharmacol. 2022 Nov 9;13:1058480. doi: 10.3389/fphar.2022.1058480 (PMC9682150; doi:10.3389/fphar.2022.1058480)

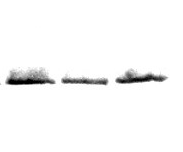

Supplement: Supplementary file 1 [file DataSheet1.ZIP › original western blot images/GLUT2.tif]

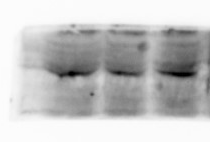

Supplement: Supplementary file 1 [file DataSheet1.ZIP › original western blot images/Ga┴.tif]

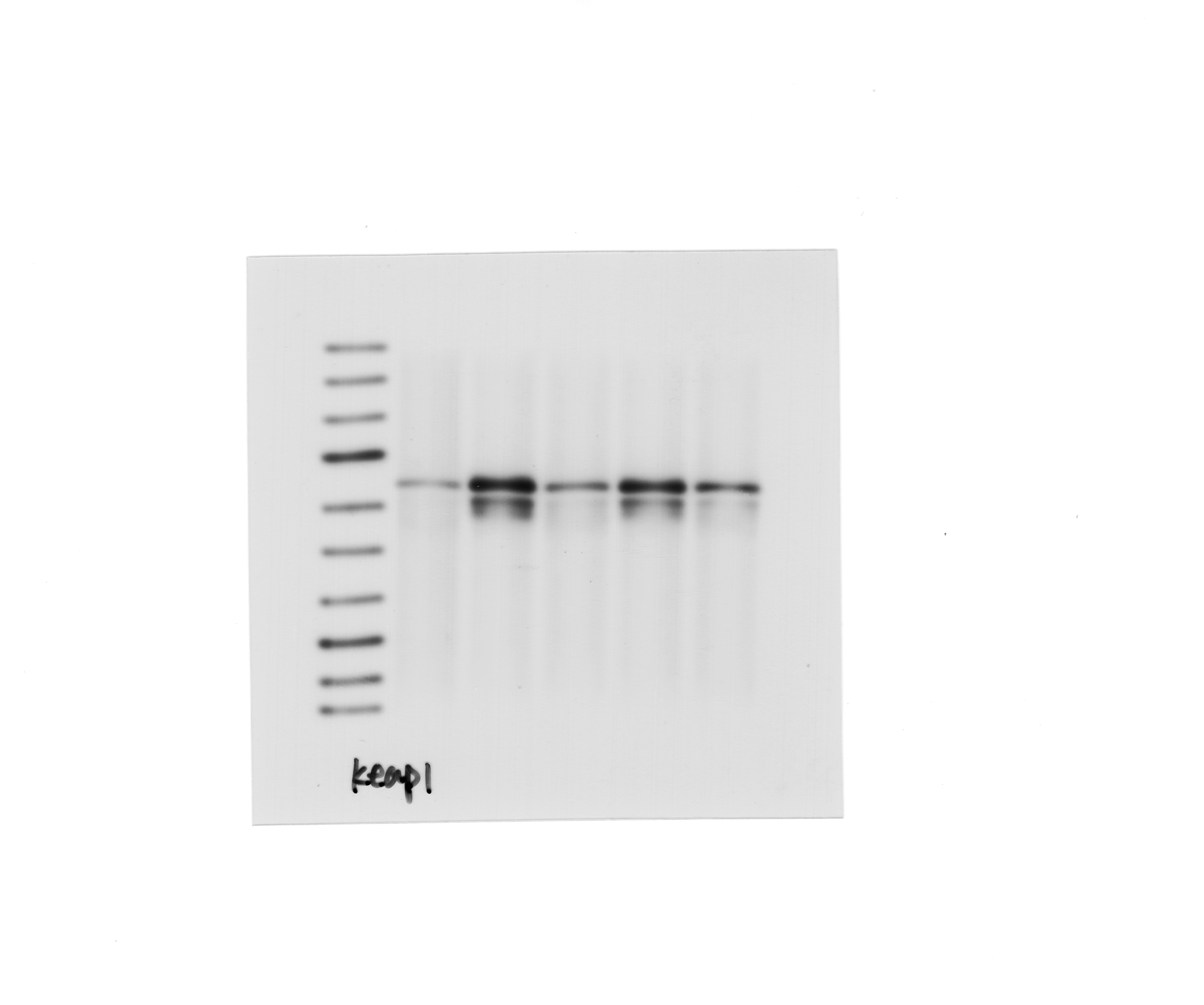

Supplement: Supplementary file 1 [file DataSheet1.ZIP › Original western blot images/Original western blot images/keap1.tif]

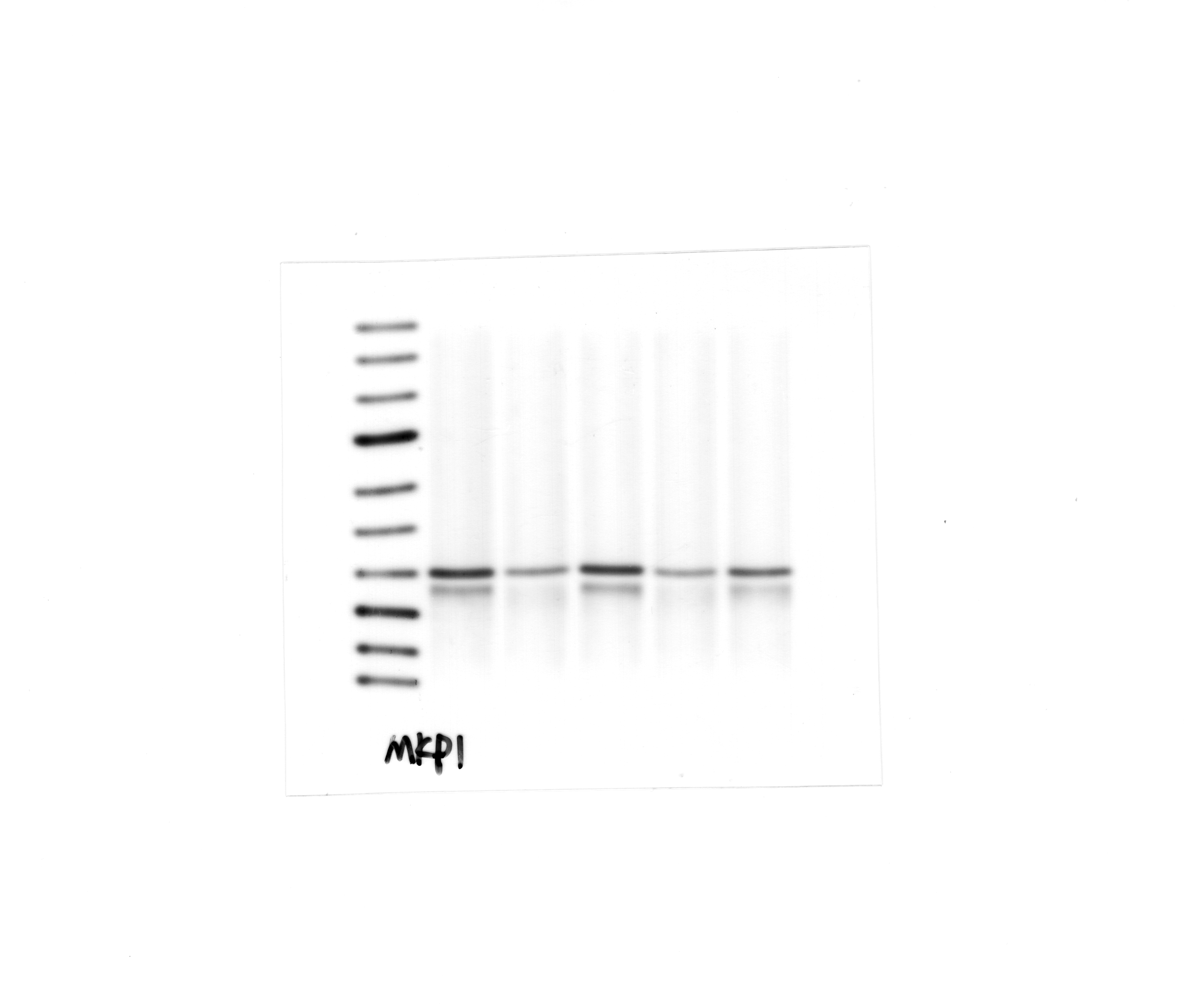

Supplement: Supplementary file 1 [file DataSheet1.ZIP › Original western blot images/Original western blot images/MKP1.tif]

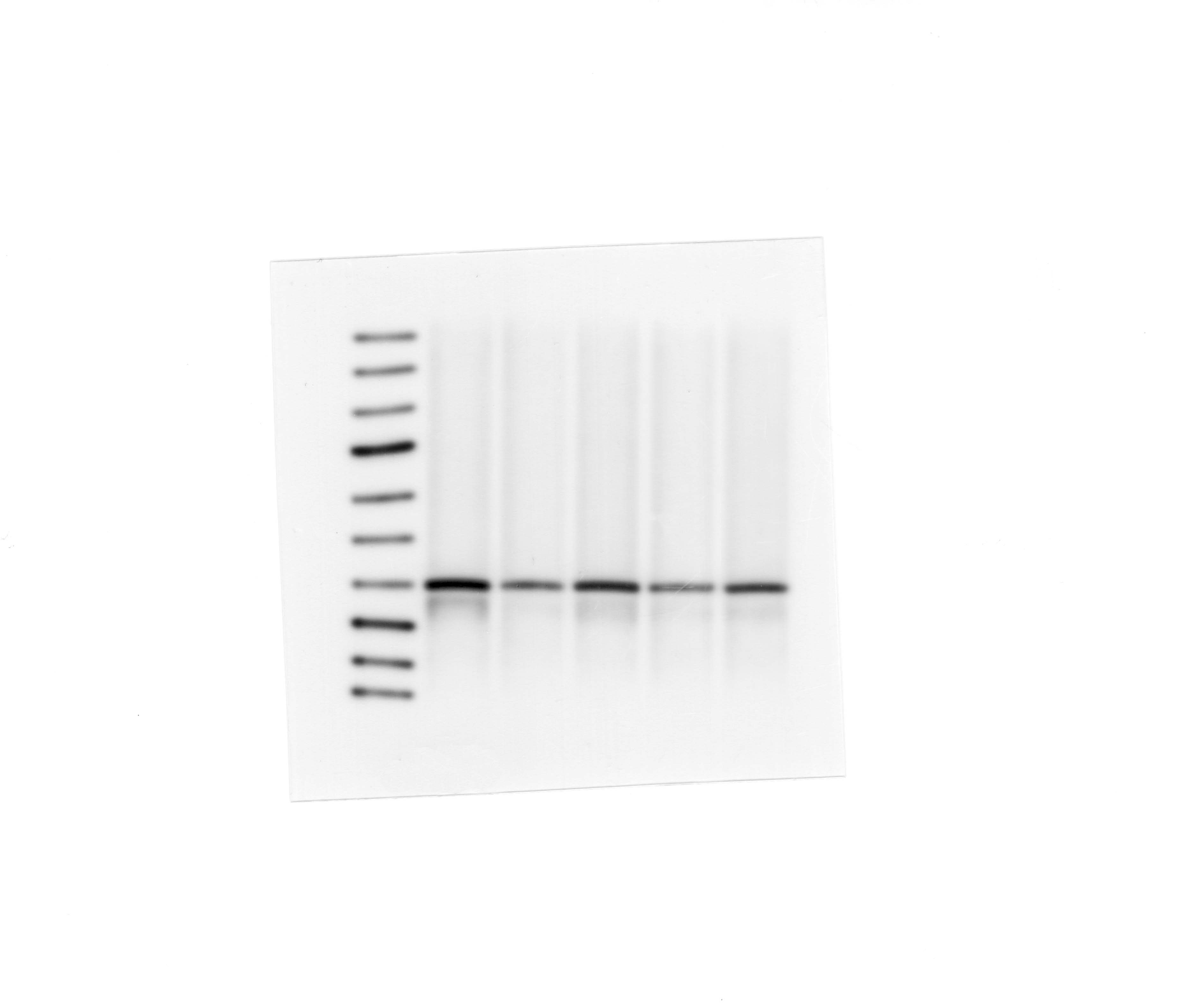

Supplement: Supplementary file 1 [file DataSheet1.ZIP › Original western blot images/Original western blot images/Nrf2.tif]

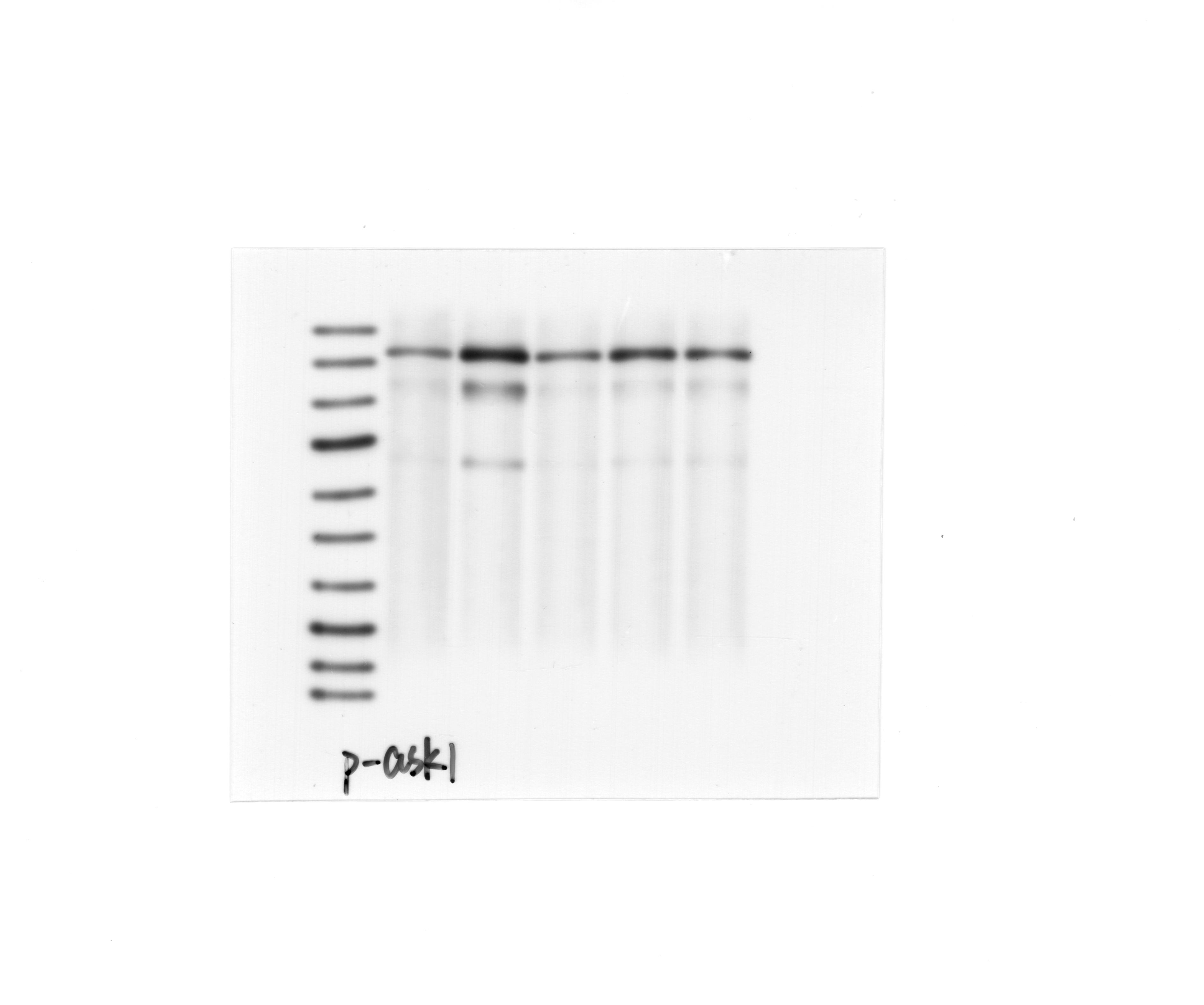

Supplement: Supplementary file 1 [file DataSheet1.ZIP › Original western blot images/Original western blot images/p-ASK1.tif]

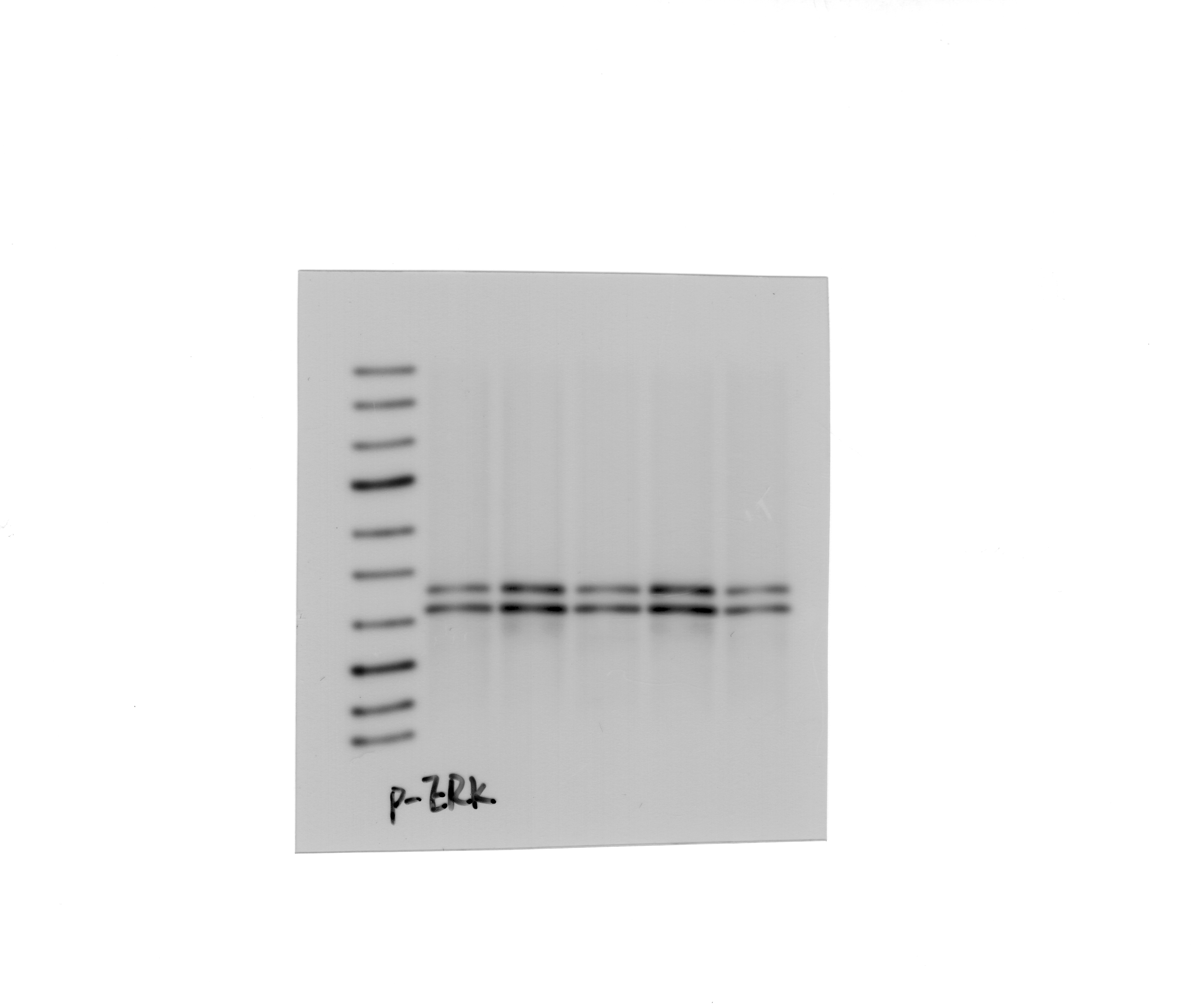

Supplement: Supplementary file 1 [file DataSheet1.ZIP › Original western blot images/Original western blot images/p-ERK.tif]

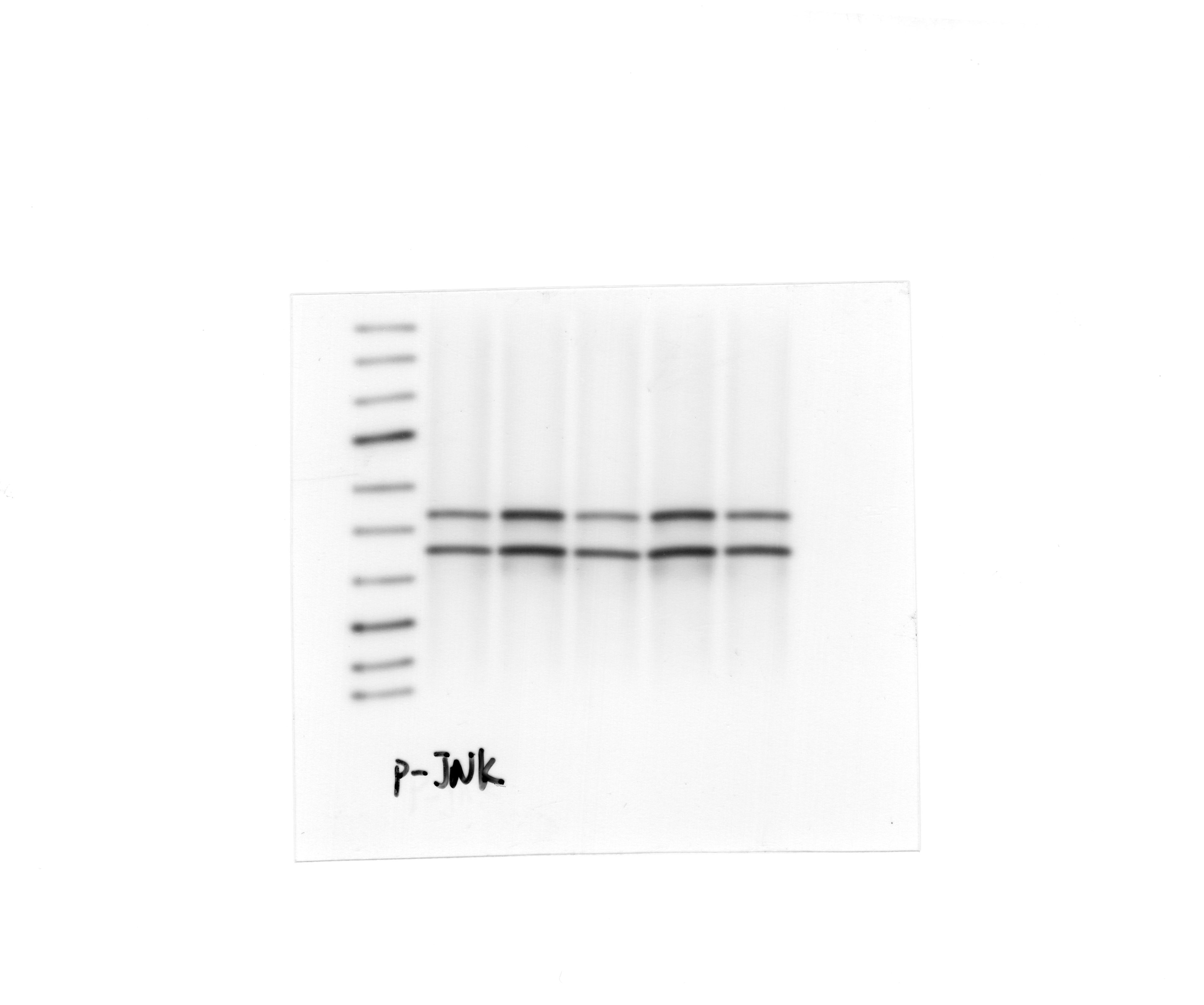

Supplement: Supplementary file 1 [file DataSheet1.ZIP › Original western blot images/Original western blot images/p-JNK.tif]

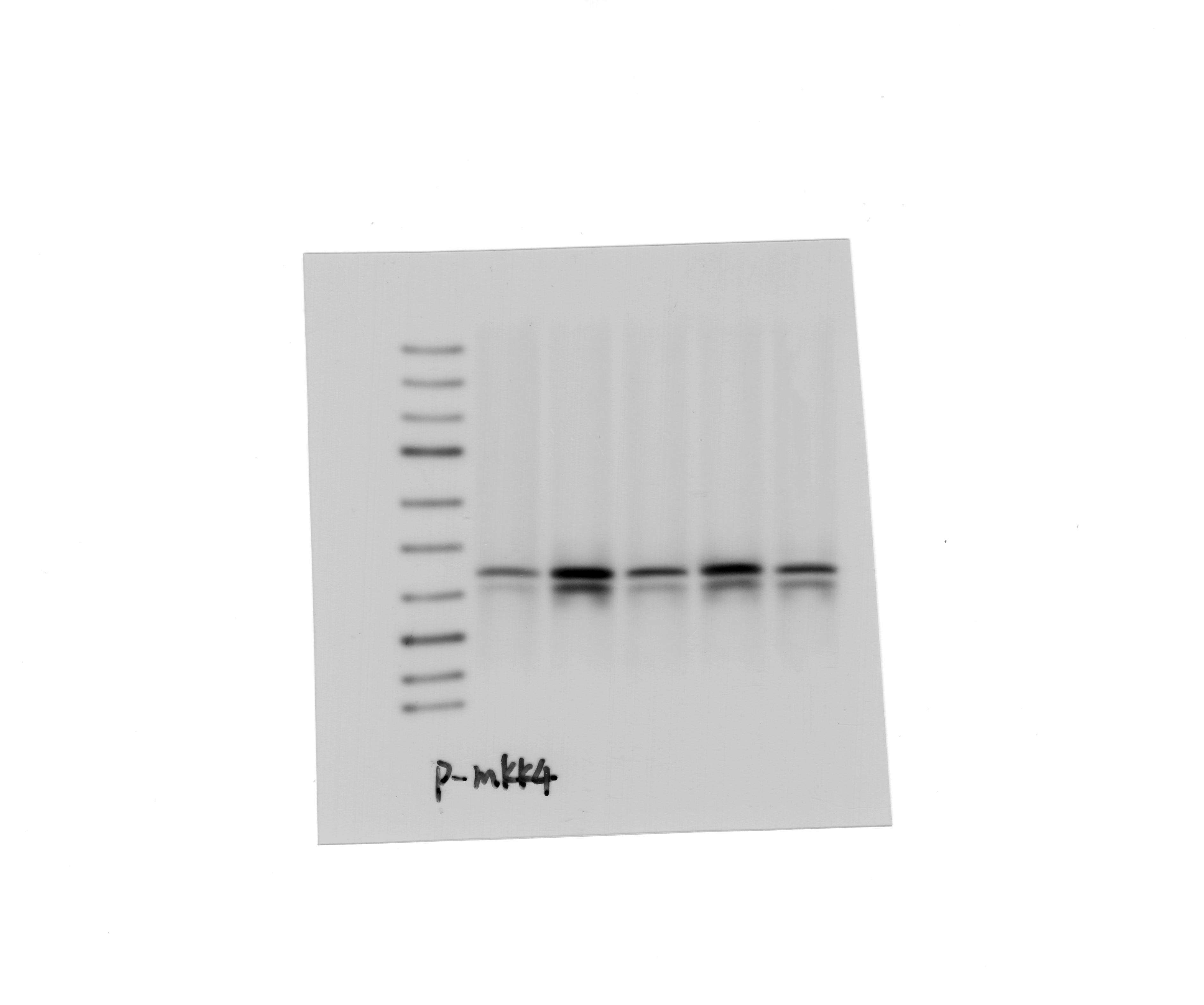

Supplement: Supplementary file 1 [file DataSheet1.ZIP › Original western blot images/Original western blot images/p-MKK4.tif]

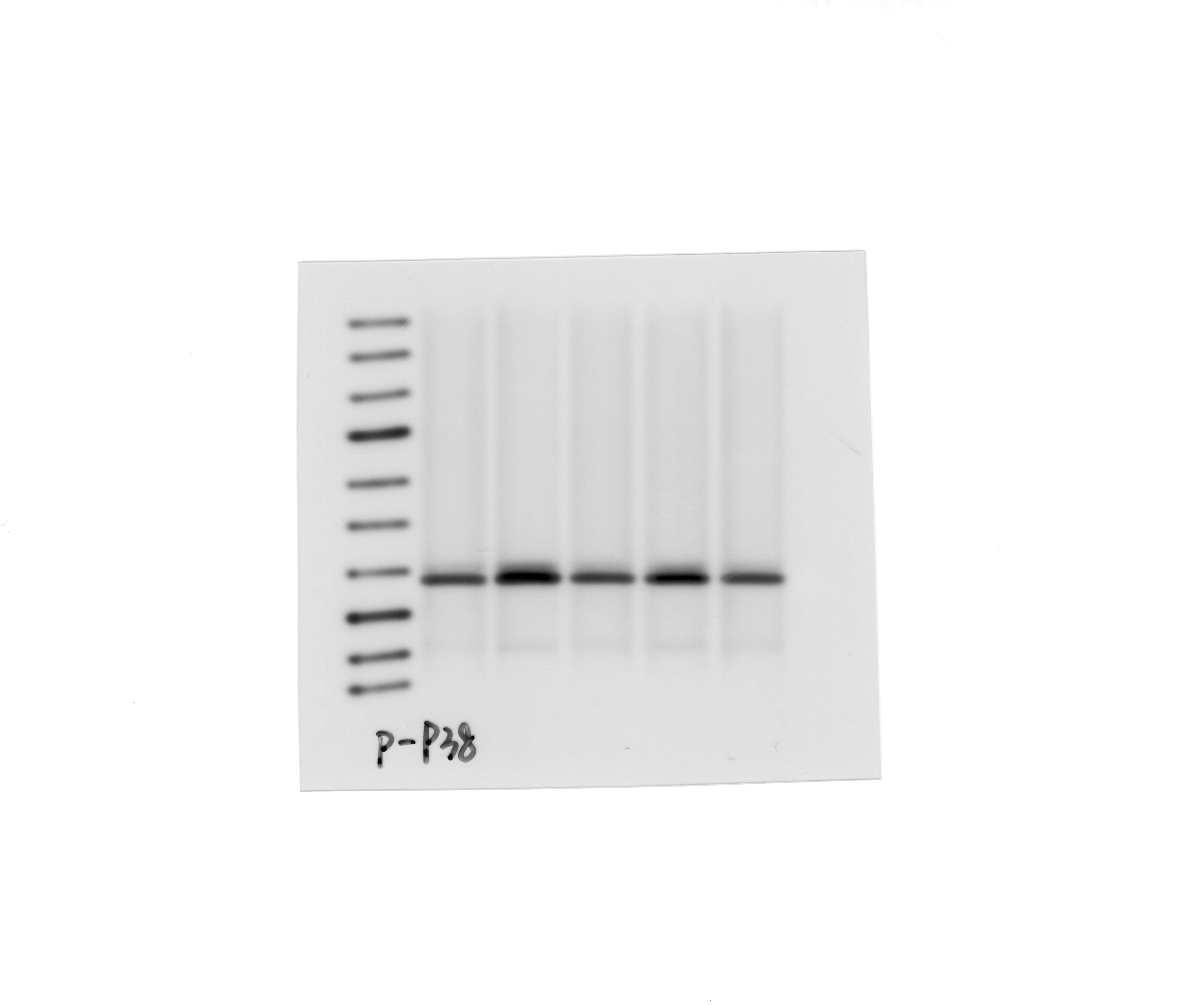

Supplement: Supplementary file 1 [file DataSheet1.ZIP › Original western blot images/Original western blot images/p-p38.tif]

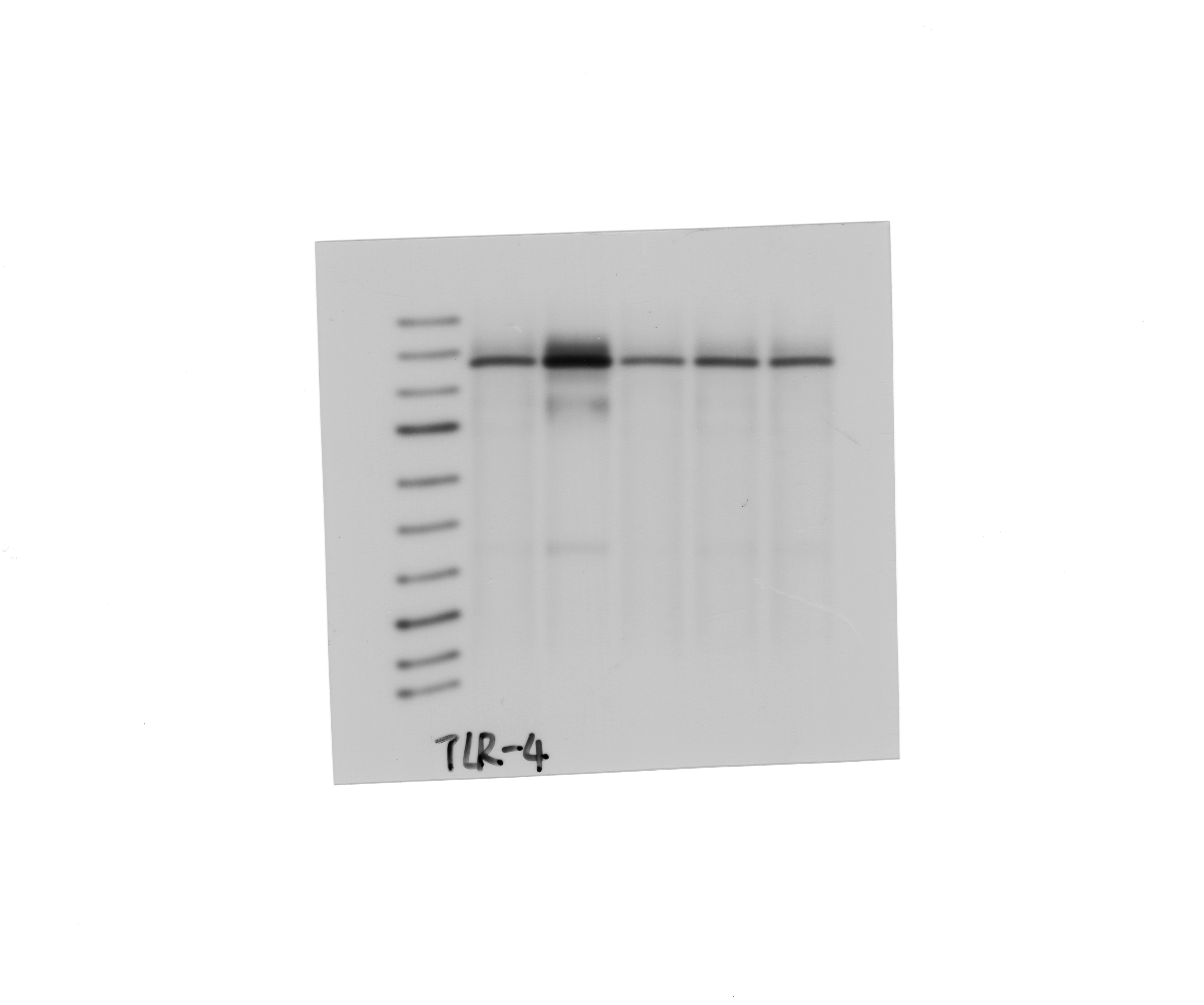

Supplement: Supplementary file 1 [file DataSheet1.ZIP › Original western blot images/Original western blot images/TLR-4.tif]

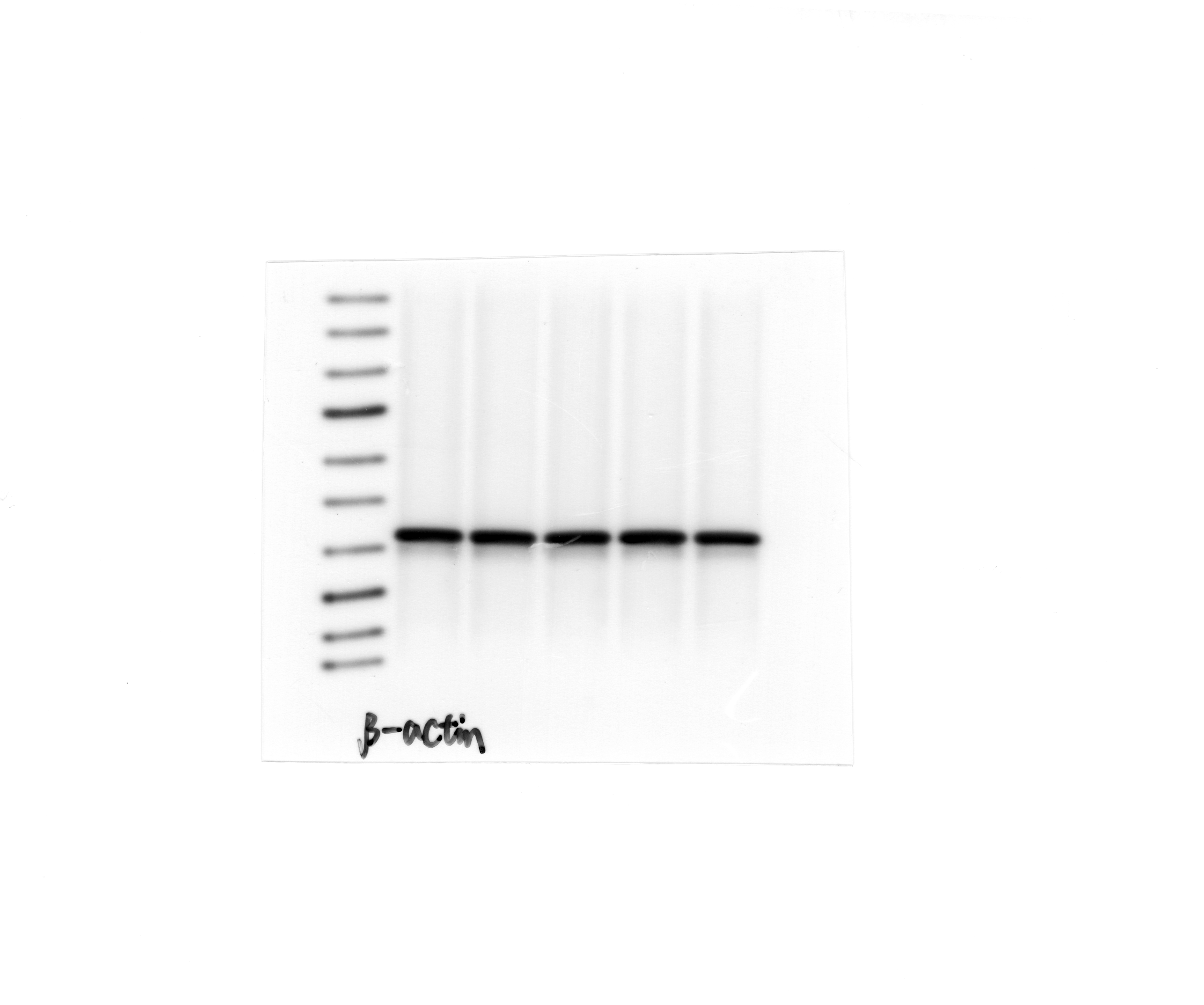

Supplement: Supplementary file 1 [file DataSheet1.ZIP › Original western blot images/Original western blot images/β-actin.tif]

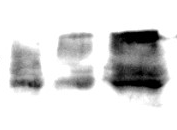

Supplement: Supplementary file 1 [file DataSheet1.ZIP › original western blot images/T1R2.tif]

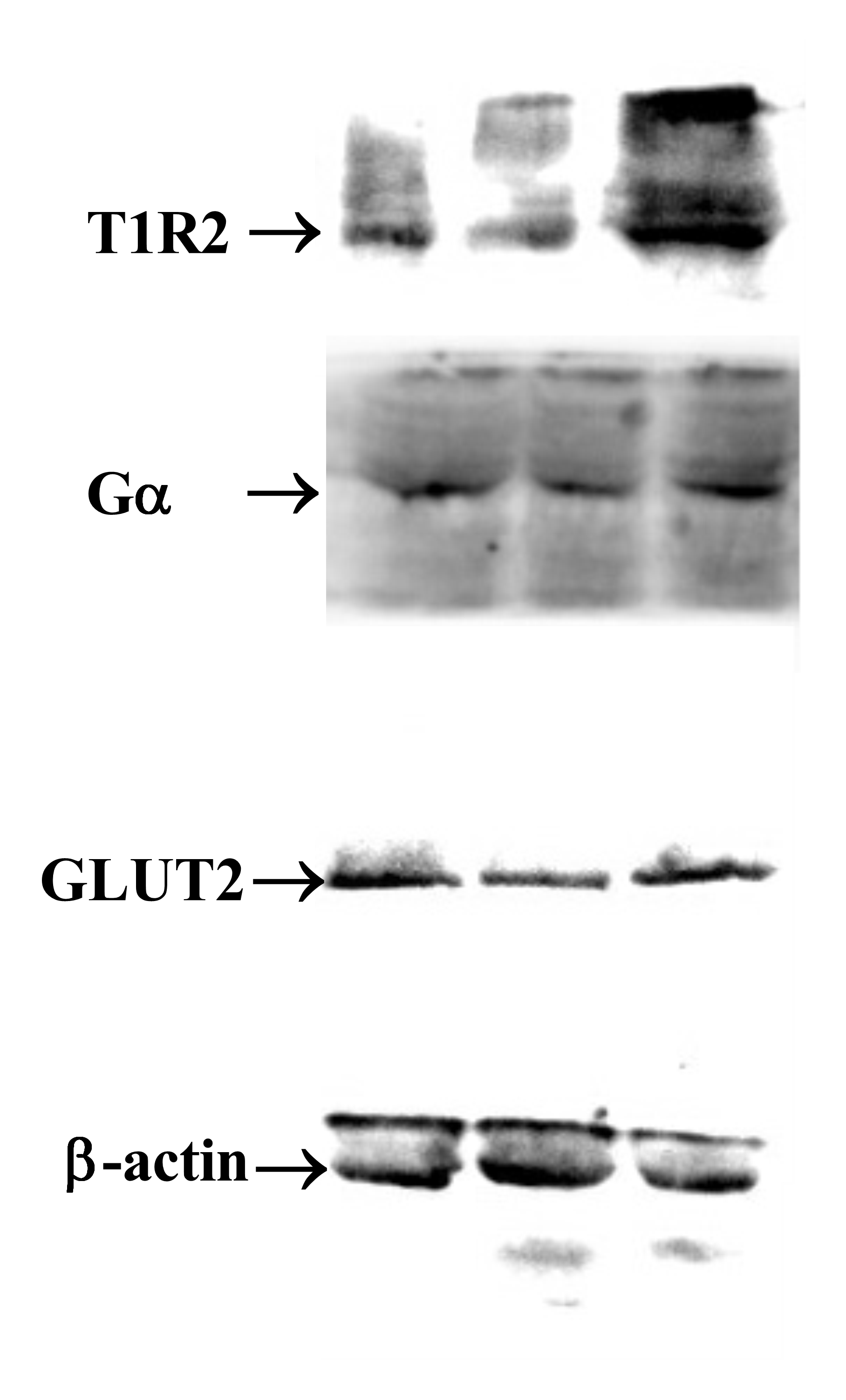

Supplement: Supplementary file 1 [file DataSheet1.ZIP › original western blot images/WB origin figure.tif]

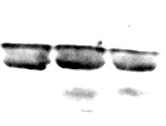

Supplement: Supplementary file 1 [file DataSheet1.ZIP › original western blot images/a┬-actin.tif]
